# PGRN inhibits CD8^+^T cell recruitment and promotes breast cancer progression by up-regulating ICAM-1 on TAM

**DOI:** 10.1007/s00262-024-03655-z

**Published:** 2024-03-30

**Authors:** Ting Zhou, Husun Qian, Dian Zhang, Wenli Fang, MengLi Yao, He Shi, Tingmei Chen, Chengsen Chai, Bianqin Guo

**Affiliations:** 1 Key Laboratory of Clinical Laboratory Diagnostics (Ministry of Education), College of Laboratory Medicine, Chongqing Medical University, Chongqing, 400016 People’s Republic of China; 2 Chongqing Key Laboratory of Translational Research for Cancer Metastasis and Individualized Treatment, Chongqing University Cancer Hospital, Chongqing, 400030 People’s Republic of China

**Keywords:** Breast cancer (BCa), Progranulin (PGRN), Tumor-associated macrophages (TAMs), Programmed cell death protein 1 (PD-1), Intercellular cell adhesion molecule-1 (ICAM-1)

## Abstract

**Background:**

Tumor microenvironment actually reduces antitumor effect against the immune attack by exclusion of CD8^+^T cells. Progranulin (PGRN) is a multifunctional growth factor with significant pathological effects in multiple tumors; however, its role in immunity evasion of breast cancer (BCa) is not completely understood.

**Methods:**

We depleted GRN (PGRN gene) genetically in mice or specifically in PY8119 murine BCa cell line, and mouse models of orthotopic or subcutaneous transplantation were used. Chimeric mice-deficient of PGRN (Grn^−/−^) in bone marrow (BM) compartment was also generated. Association of PGRN expression with chemokine production or BCa development was investigated by histological and immunological assays.

**Results:**

We found PGRN was involved in exhaustion of cytotoxic CD8^+^T cell in BCa with the increasing expressions of M2 markers and intercellular cell adhesion molecule-1 (ICAM-1) on macrophages. Specifically, ablation of PGRN in PY8119 cells reduced tumor burden, accompanied by the infiltrating of cytotoxic CD8^+^T cells into tumor nests. Moreover, our result revealed that blockade of PD-1 in PGRN-depleted tumors exhibited better antitumor effect in vivo and significantly decreased tumor burden.

**Conclusion:**

These findings suggest that inhibition of PGRN may act as a potential immune-therapeutic strategy by recovering infiltration of CD8^+^T cell in BCa tissue and thereby enhancing the response to anti-PD-1 therapy.

**Supplementary Information:**

The online version contains supplementary material available at 10.1007/s00262-024-03655-z.

## Introduction

CD8^+^ cytotoxic T cells, also known as cytotoxic T cells (CTL), are vital in the immune response against cancer [1] and closely related to a good clinical prognosis in many tumor types, including ovarian, colon, breast, and pancreatic cancer [2–6]. In recent years, it has had a positive effect when immune checkpoint inhibitors are used in clinical treatment for many types of cancers, with anti-programmed cell death protein 1 (PD-1) inhibitors being one of the leading candidates [7]. However, the efficacy of immune checkpoint inhibitors depends on the infiltration rate of CD8^+^T cells in tumors. Therefore, it is necessary to reveal the potential mechanism that suppressing CD8^+^T cells infiltration. CD8^+^T cells are poorly observed in breast cancer tissues, and thereby using immune checkpoint inhibitors alone did not show a good clinical outcome in patients with breast cancer [8–11]. Breast cancer is characterized by extensive immunosuppressive cell infiltration and fibrosis, which all together critically promote the disease progression [12–14] and its response to therapy [15]. The tumor micro-environment (TME) is also seen as a major barrier to CD8^+^T cell infiltration in breast cancer [10, 16], and it is necessary to overcome this defect for the successful use of immune checkpoint inhibitors [17, 18].

Tumor-associated macrophages (TAMs) are the most abundant and important immune cell types in breast cancer. TAMs have shown their prognostic significance in BC, and their infiltration rate correlates with tumor progression and metastasis [19, 20]. Depending on the activation signals, macrophages can be polarized into M1-like inflammatory macrophages or into M2-like anti-inflammatory, immunosuppressive macrophages [17, 20, 21]. Previous evidence suggested that TAMs were engaged in complex network interactions with cancer stromal cells, which accelerated the formation of the immunosuppressive TME [22, 23], and the recruitment of immunosuppressive cell into tumors led to the decrease of CD8^+^T cell infiltration and the increase of tumor burden [17, 20, 22, 24–28]. However, the mechanism by which macrophages regulate T cell infiltration is still not disclosed.

Progranulin (PGRN) is an 88-kDa glycoprotein (GP88) and expressed mainly in neurons and immune cells [29]. PGRN plays a role in a variety of biological processes such as tissue remodeling and repair during embryonic development, wound repair, and inflammation [30–32]. Interestingly, numerous studies have demonstrated that PGRN is also expressed in tumor cells, including breast cancer, and correlates with the tumor progression, metastasis, and drug resistance [33, 34]. Overexpression of PGRN has been observed in clinical specimens of breast cancer, especially in cancers with high malignancy [35, 36]. Yet, whether and how PGRN affects tumorigenesis and immune escape in the breast cancer remains unknown.

We have recently identified that PGRN promoted CD8 + T cells exclusion through the interaction of PD-L1 on TAMs with PD-1 on CD8 + T cells, which also inhibited the proliferation and activation of CD8 + T cells [37]. However, the immunosuppressive effects of PGRN on the TME are multifaceted, and other mechanisms by which PGRN affected CD8^+^T cells infiltration and function remain unexplored. Hereby, in this study, we firstly revealed that tumor-derived PGRN was a key player that contributed to CD8^+^T cell exclusion and the resistance to anti-PD-1 treatment. Specifically, tumor-derived PGRN promoted the polarization of TAMs to M2 macrophages, and then, PGRN-treated TAMs inhibited proliferation and activation of CD8 + T cells via ICAM-1-mediated interaction with CD8^+^T cells in BCa tumor microenvironment. On the other hand, we showed that depletion of PGRN alleviated CD8^+^ T cell exclusion in BCa tissue and enhanced the response to anti-PD-1 treatment. Overall, our study suggests that PGRN may serve as a potential therapy target for breast cancer.

## Materials and methods

### Mice

Six–eight weeks’ old wild-type (WT) female C57BL/6 were purchased from Chongqing Medical University (Chongqing, China), and Grn^−/−^mice were generously gifted from Dr. Yibing Yin. Mice from both stains were maintained in the pathogen-free condition. All animal experiments were performed in accordance with approved protocols by the Institutional Animal Care and Ethical Committee of Chongqing Medical University.

### Cells

Murine breast cancer cells PY8119 (RRID:CVCL_AQ09) were cultured in F12/DMEM (1:1) with 10% fetal bovine serum (FBS) and 1% penicillin/streptomycin. To generate the stable PY8119/Grn^−/−^ cells, CRISPR technology was used. Ablation of the GRN gene mediated by CRISPR-Cas9 was acquired with transposon plasmid (provided by Haixing Bioscience) containing expression cassettes for hSpCas9, Puro and chimeric guide RNA. To target exon 2 ~ exon 13 of the GRN gene, two guide RNA sequence of CCCCGGGCCTTTTGAAGGGCTGG and GTAGGCCGGGGAGACGTTAGGGG were selected through the http://crispr.mit.edu website. Plasmid containing the guide RNA sequence was electro-transfected into cells using Neon transfection system according to the manufacturer’s instructions (Thermo Fisher Scientific). After twenty-four hours’ post-transfection, cells were selected by puromycin (2ug/mL). After three days, single colonies were transferred into 96-well plates. To determine the presence of insertions or deletions (indels) in GRN targeted clones, genomic DNA was isolated using a Blood/Cultured cells DNA Kit (Simgen, 3,002,050). And GRN exon 2 ~ exon 13 PCR amplification was acquired using 2 × Taq Master Mix (Dye Plus; Vazyme, P112) of primers flanking exon; Forward: 5’-CTTGCTGCACACTGGTAGGA-3’; Reverse: 5’-GGGCGATCCTGGAGACTAGA-3’. PCR product was cloned into Mighty TA-cloning Kit, (Takara, 6028) and transformed into E. coli DH5α competent cells (Thermo Fisher Scientific). Plasmids were isolated from 8–10 single colonies and sequenced by Sanger sequencing (GENEWIZ, China). Clones with mutations in both alleles were selected for downstream studies. Cell lines were generated using the above strategy including PY8119 GRN^+/+^, PY8119 GRN^−/−^A, PY8119 GRN^−/−^B, PY8119 GRN^−/−^C and PY8119 GRN^−/−^ cells. All clones were maintained under the same conditions as parental cells.

Murine breast cancer cells 4T1 and Murine macrophages Raw267.4 were cultured in DMEM with 10% fetal bovine serum (FBS) and 1% penicillin/streptomycin.

### In vivo animal studies

A total of 5 × 10^6^ PY8119 cells suspended in 100 μl of PBS were injected into the fourth right mammary fat pad of WT or Grn^−/−^ C57BL/6 mice. For PY8119 and PY8119 Grn^−/−^ primary tumor growth, 1 × 10^6^cells in 100 μl of PBS were injected subcutaneously in right flank of the mice. Mice were randomized into groups by body weight. Tumor sizes were monitored every other day using an electronic caliper. Tumor growth was evaluated by measuring tumor volume (TV = 0.5 × length × width^2^) every 3 days until they were eliminated 30 days after treatment. For selected experiments, macrophages were depleted using Clodronate liposomes (LIPOSOMA) or CSF-1R inhibitor (Selleckchem; Ki20227). Clodronate liposomes were administered via i.p. injection daily with 100ul/10g; PBS liposomes (LIPOSOMA) were used as control. CSF-1R inhibitor Ki20227 was administered daily at a concentration of 20mg/kg in 20% Captisol (Ligand Pharmaceuticals) via i.p. injection. Captisol (20%) was used as vehicle control. For immune checkpoint blockade, PD-1 antagonist (BioXCell; clone RMP-1) or Rat IgG2 (BioXCell; clone 2A3) isotype control was administered every 3 days by i.p. injection at 250 mg/dose for a total of 4 injections in 20 days (early phase treatment, d3).

### Bone marrow transplantation

Thirty WT and Thirty Grn^−/−^ female C57BL/6 mice were prepared. Bone marrow transplantation was performed by reconstituting the bone marrow of lethally irradiated (8.5 Gy) female and 6-week-old C57BL/6 mice by injecting with 5 × 10^6^ total bone marrow cells isolated from Grn^−/−^mice or WT mice. After 4 weeks, engraftment of Grn^−/−^bone marrow was assessed by genomic DNA PCR according to The Jackson Laboratory’s protocol on peripheral blood cells from fully recovered bone marrow-transplanted mice. After confirmation of successful bone marrow reconstitution, mice were enrolled in tumor studies.

### Peritoneal macrophages isolation

Peritoneal macrophages were generated according to previous literature [38]. Briefly, each C57BL/6 mouse was injected intraperitoneally with 1 ml of 3% thioglycollate (Difco) on day 1. After administration, the mice were kept under daily observation to check for abnormal activities. Mice were killed 4 days after injection. Peritoneal macrophages were harvested from peritoneum, after being injected with 7-ml cold PBS into peritoneal cavity. The peritoneal cells were centrifuged at 1500 RPM for 10 min and then seeded into cell culture dishes. The suspension cells were discarded by being washed with PBS after 2 h. And the adherent cells were considered as peritoneal macrophages.

### Preparation of conditioned media

Conditioned medium (CM) was acquired from 4T1 cells. Briefly, the medium was removed from 70% confluent cells, and the cells were washed 3 times with PBS before addition of serum-free medium. Cells were incubated for 24 h in serum-free medium and then collected and filtered through 0.45-mm filters before use.

### Flow cytometry and cell sorting

Single-cell suspensions from murine tumor tissues were prepared by mechanical and enzymatic disruption in PBS with 1 mg/ml collagenase D and 4 μg/ml DNase I for 1 h in 37 °C with periodic vortexing. Cells were filtered through a 70-um cell strainer, resuspended in PBS and centrifuged at 500 g for 5 min, followed by the removement of the red blood cells (RBCs) with RBC lysis buffer. The cells were blocked by 0.5% BSA for 30 min on ice with FC block and then stained with the following antibodies separately: PerCP/Cyanine5.5 anti-mouse CD45 (103,131; BioLegend), APC/Cyanine7 anti-mouse F4/80 (123,117; BioLegend), FITC anti-mouse CD3 (100,203; BioLegend), PE anti-mouse CD69 (104,507; BioLegend), APC anti-mouse CD206 (141,707; BioLegend), PE anti-mouse CD86 (105,007; BioLegend). APC anti-mouse CD8a (100,711; BioLegend). PE anti-mouse IFN-*γ* (505,807; BioLegend), PE anti-mouse PD-1 (135,205; BioLegend) and PE anti-mouse Ki67 (652,403; BioLegend). For intracellular staining, cells were first fixed (eBioscience, IC fixation buffer) and permeabilized (eBioscience, 1 × permeabilization buffer).

### In vitro T cell activation assay

Primary splenocytes were obtained from C57BL/6 mice. Dissected spleens were dissociated in PBS and passed through a 70-um cell strainer to obtain a single cell suspension. Cells were centrifuged, and red blood cells were lysed using RBC lysis buffer. Obtained splenocytes were cultured in RPMI supplemented with 10% FBS. For T cell activation assays, splenocytes were stimulated using Mouse T-Activator CD3a and CD28 (BioLegend). The splenocytes activated (S) were then cocultured with PMs from WT and Grn^−/−^mice (4:1 ratio, S:M). Cells were plated in 48-well plates and incubated at 37℃ for 24 h. Subsequently, Brefeldin A (MCE; 1:1000) was added to the cells for 5 h. Cells were then harvested and stained with CD8 (BioLegend), IFNγ (BioLegend) and CD69 antibodies and they were analyzed by flow cytometry. For some experiments, mouse ICAM-1 neutralizing antibody (GY20190816; Novogen; 5 mg/mL) were used.

### In vitro T cell proliferation assay

For T cell proliferation assay, splenocytes derived from C57BL/6 mice were labeled with 5 mmol/L carboxyfluorescein diacetate succinimidyl ester (CFSE; BioLegend) and incubated for 10 min at 37 °C in the dark. Cells were then resuspended in RPMI 1640 supplemented with 10% FBS and stimulated with Mouse T-Activator CD3a and CD28. Activated splenocytes (S) were then cocultured with PMs from WT and Grn^−/−^mice (4:1 ratio, S:M). Cells were plated in 48-well plates and incubated at 37 °C for 72 h. Subsequently, cells were harvested and stained with CD8 antibody. Proliferating CD8^+^T cells were tracked by flow cytometry.

#### Immunofluorescence

Slides from tumors were deparaffinized in xylene and dehydrated in graded ethanol solutions. The sections were blocked in goat serum blocking solution for 1 h at room temperature. The slides were incubated overnight at 4 °C with the following antibodies for multicolor immunofluorescence staining: F4/80 (Cell Signaling Technology, 30,325 T, 1:200), CD206 (Proteintech, 18,704–1-AP, 1:200), Arg1 (Abcam, ab96183, 1:100), iNOS (Abcam, ab178945, 1:200), CD8 (Bioss, bs0648R, 1:100), aSMA (Cell Signaling Technology, 19,245, 1:200); Ki67 (Cell Signaling Technology, 12,075, 1:100); CSF1R (Abcam, ab254357, 1:100) and Granzyme B (Cell Signaling Technology, 17,215, 1:200). Immunofluorescence staining was detected with fluorescence microscope.

#### Immunohistochemistry analysis

Sections from tumors were cut into 5μm in thickness and deparaffinized in xylene for 15 min. Slides were dehydrated in graded ethanol solutions for 5min. Tissue sections were incubated overnight at 4 °C with primary antibodies: CD8 (Cell Signaling Technology, 85,336, 1:100); CD68 (Abcam, ab25333, 1:100); PGRN (Proteintech, 18,410–1-AP, 1:100). Second-HRP-conjugated antibodies were incubated for 30 min at room temperature. IHC staining images were examined with microscopy.

#### Western blot

The cells were lysed in RIPA lysis buffer (Beyotime, China, P0013B) supplemented with protease inhibitors PMSF (Beyotime, China, ST506). Protein concentration was determined by using BCA protein assay kit (Beyotime, China, P0012S). The primary antibodies included ICAM-1 (Abcam, ab178945, 1:1000), PGRN (R&D Systems, MAB25571, 1:50) and GAPDH (Proteintech, 66,009–1-Ig, 1:2000).

#### TUNEL assay

The frozen slices from tumors were restored in an oven at 37 °C for 10-20min and fixed in 4% paraformaldehyde for 30min. Protease K working solution (protease K:PBS = 1:9) and 0.1% triton in turn were added to cover the tissue, then take appropriate amount of TDT enzyme, dUTP and buffer in the Tunel kit (Servicebio) according to the number of slices and tissue size and mix at 1:5:50 ratio, and added to cover the tissue incubated at 37 °C for 1 h in a flat wet box. TUNEL stainings were examined with fluorescence microscope.

#### CCK-8 assay

5 × 10^3^ viable PY8119^WT^ cells or PY8119^KO^ cells were seeded in 96-well plates. After culture for 24 h, CCK-8 reagent was added to assay plates. Following incubation for 24 h, 48 h, 72 h at 37 °C, the absorbance values were measured by microplate reader.

#### Statistical analysis

Unpaired Student’s t test was adopted for mean difference comparison between two groups. One-way ANOVA followed by multiple comparison was adopted for multiple groups. All data were presented as mean ± SEM (standard error of mean). *P* < 0.05 was considered as significant. All the experiments were performed independently for three times.

## Results

### PGRN promoted tumor growth by inducing CD8 + T cell exclusion in breast cancer

**Fig. 1:**
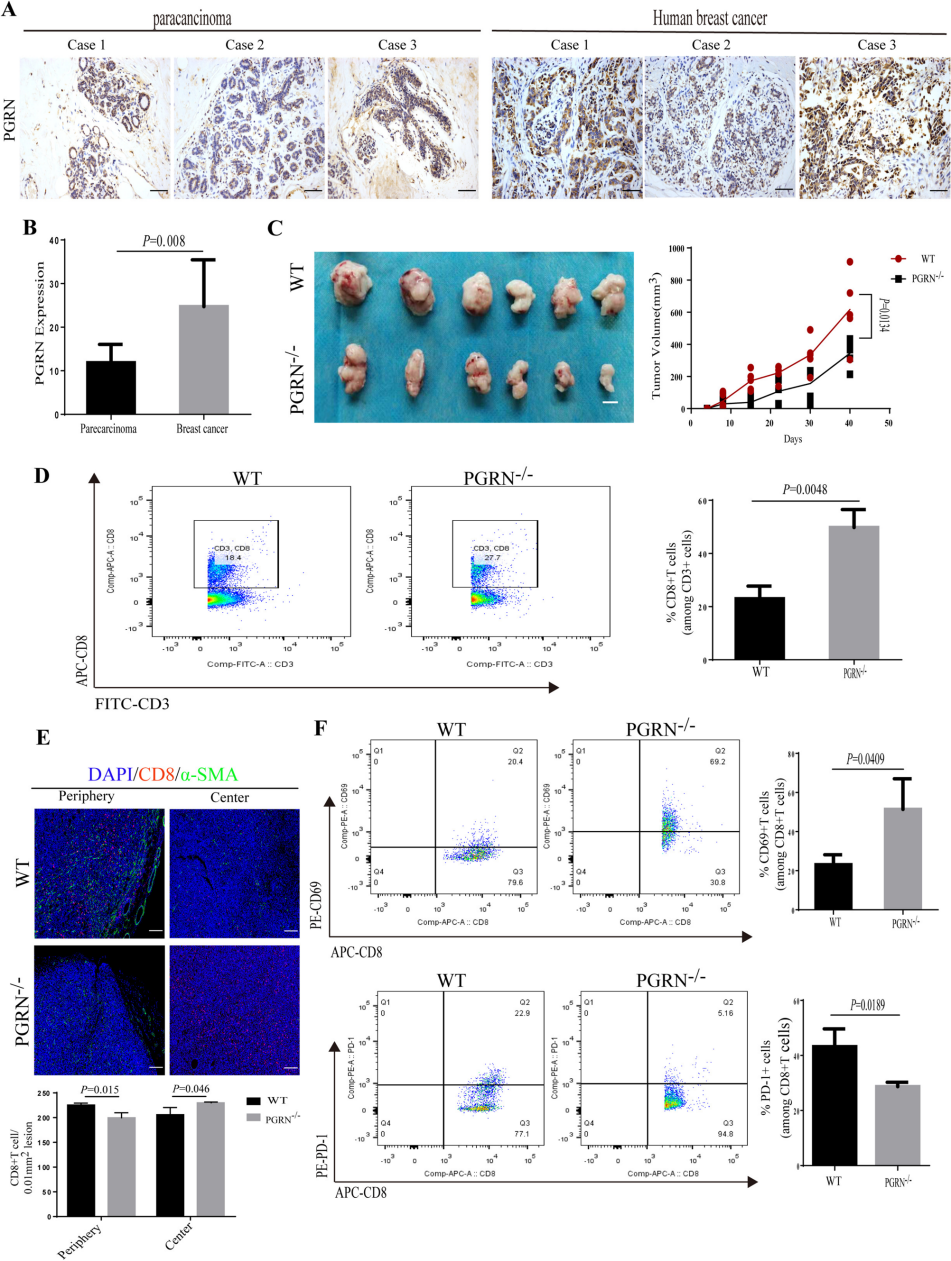
PGRN promoted tumor growth by inducing CD8^+^T cell exclusion in BCa tissue. **A** IHC images of PGRN level in human breast cancer and paracancerous tissue sections (*n* = 68); scale bar, 50um. **B** Quantification of PGRN level in human breast cancer and paracancerous tissue sections (*n* = 68). **C** Breast cancer PY8119 cells were injected in situ into the fat pads of C57 wild-type mice and PGRN knock-out mice (*n* = 6 per group) and tumor volume curve; scale bar,10mm. **D** Percentage of CD8^+^ T cells over time assessed by flow cytometry analysis. **E** Representative images of MxIHC staining in tumors from WT and PGRN^−/−^mice; scale bar, 100um. **F** Percentage of CD69^+^CD8^+^T cells and PD-1^+^CD8^+^T cells over time assessed by flow cytometry analysis

Previously we revealed that PGRN promoted CD8 + T cells exclusion through the interaction between PD-L1 on TAMs with PD-1 on CD8 + T cells, which inhibited the proliferation and activation of CD8 + T cells [37]. PGRN was expressed in both breast cancer cells and non-cancer cells (e.g. immune cells) [29]; however, the cellular source of CD8 + T cell-suppressing PGRN in BCa tissue was still not clarified. To address this, firstly, we examined the expression levels of PGRN in human breast cancer tissues and paracancerous tissues by immunohistochemistry. PGRN was significantly overexpressed in the breast cancer tissues compared to paracancerous tissues (Fig. 1A, B). Then, to exclude the effect of PGRN derived from xenograft immune system, PGRN ^WT^ mice and PGRN^−KO^ mice were used in orthotopic xenograft with PGRN ^WT^ PY8119 cells. As shown in Fig. 1C, though the tumor volume of PGRN^−/−^ mice by orthotopic xenograft was smaller compared with the PGRN WT mice, PGRN knock-off in mice did not prevent tumorigenesis of PGRN ^WT^ PY8119 cell, which hinted that PY8119-derived PGRN played a dominate role in tumor growth compared to mice-derived PGRN (Fig. 1C). Next, to investigate the mechanism by which PGRN promoted tumor growth, using flow cytometry, we found the fewer infiltrating CD8^+^T cells were displayed in the PGRN WT group compared to the PGRN^−/−^ group (Fig. 1D). Then, the localization of CD8^+^T cells was examined by immunofluorescence in WT and PGRN^−/−^ tumors. Myofibroblasts are metabolically and morphologically distinctive fibroblasts expressingα-SMA, which can be used to distinguish myofibroblasts and tumor cells, andα-SMA + cells were shown to indicate the edge of the tumor [39, 40]. The immunofluorescence results showed that PGRN remarkably altered the distribution of CD8^+^T cells, which were excluded from the tumor center (Fig. 1E). We also analyzed patient tissue sections of breast cancer tissues and paracarcinoma tissues, and CD8^+^T cells were accumulated at the tumor periphery in PGRN-rich tumor tissues (Supplementary Fig. S1). Additionally, to investigate whether PGRN influenced function of CD8^+^T cells, the expression levels of T cell activation marker CD69 and the inhibitory immune checkpoint receptor PD-1 were analyzed. CD8^+^CD69^+^PD-1^−^T cells isolated from PGRN^−/−^ group were significantly increased, whereas CD8^+^T cells derived from WT group were rarely active (Fig. 1F). Collectively, these results indicated that PGRN reduced CD8^+^T cell infiltration, and the CD8^+^ T cells with cytotoxic function were depressed.

### Macrophage inhibition by PGRN depletion increased CD8 + T cell infiltration into tumor nests

**Fig. 2:**
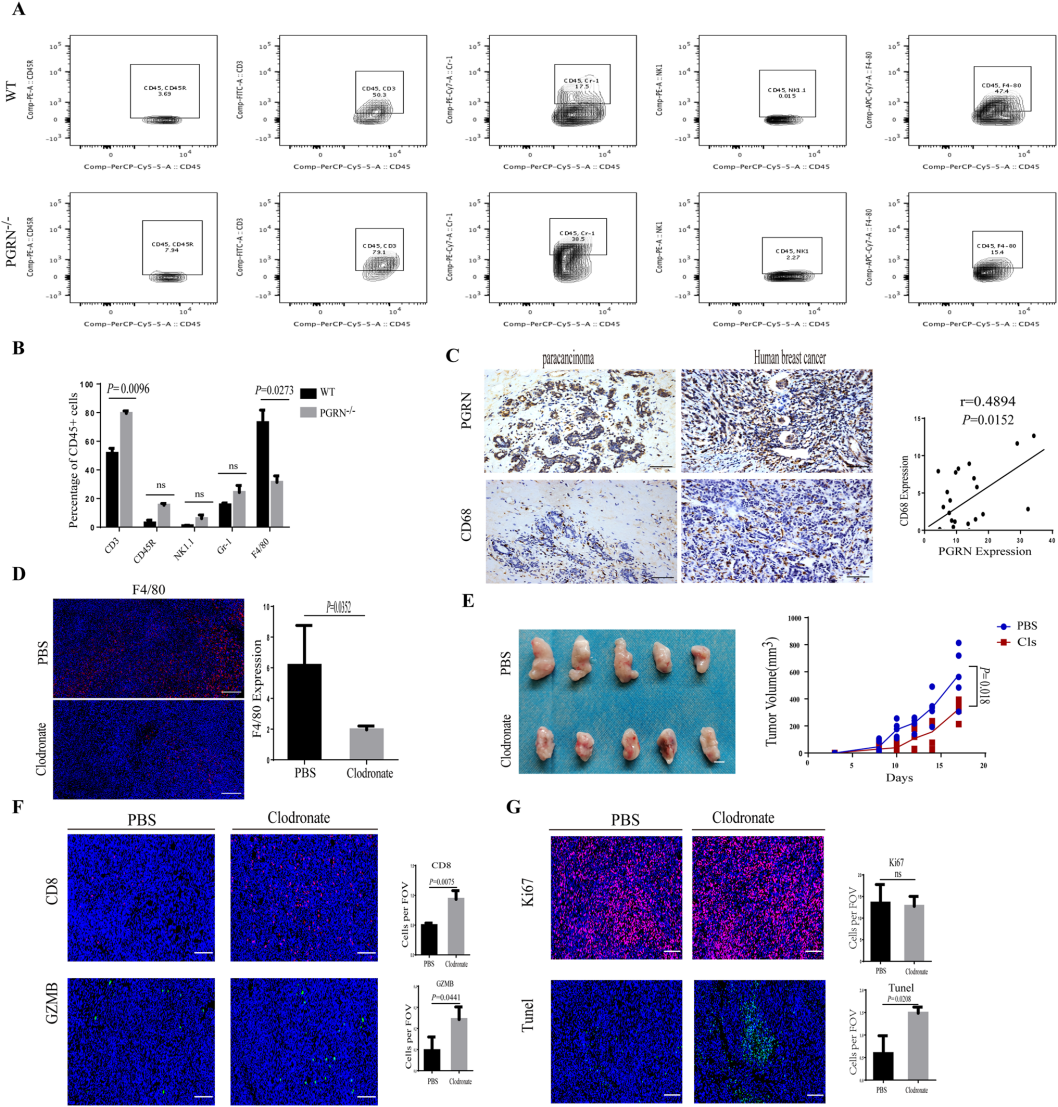
Macrophage depletion increased CD8^+^T cell infiltration into tumor nests. **A**, **B** Composition of leukocytes was shown as a percentage of CD45^+^ cells using the following definitions: B cells (CD45^+^CD45R^+^), T cells (CD45^+^CD3^+^), NK cells (CD45^+^NK1.1^+^), neutrophils (CD45^+^Gr-1^+^), TAMs (CD45^+^F4/80^+^) by flow cytometry analysis (**A**) and quantification of composition of leukocytes (**B**). **C** IHC images of PGRN^+^ cells and CD68^+^macrophages in human breast cancer and paracancerous tissue sections (*n* = 68); scale bar, 50um. Pearson correlation plots of the expression of PGRN and CD68 in human tumor samples. **D** Immunofluorescent staining and analysis of TAM (F4/80^+^) in tumors treated with liposomes containing PBS or clodronate (**D**); scale bar, 100um. **E** Tumor tissue and volume curve (*n* = 5 per group); scale bar, 10mm. **F**, **G** Representative immunofluorescent staining and quantification of CD8^+^T cells, activated T cells (GZMB^+^), Ki67^+^ and apoptotic cells (Tunel^+^) frequency in tumor treated with liposomes containing PBS or clodronate; scale bar, 50um

To clarify the effect of PGRN on immune cells in BCa tissue, the immune cell composition of PGRN WT and PGRN^−/−^tumors was analyzed by flow cytometry, respectively. It showed that in the PGRN^−/−^ group fewer tumor-associated macrophages (TAMs; CD45^+^F4/80^+^) were identified; conversely, T cells (CD45^+^CD3^+^) were significant increased, but there were no significant differences in B cells (CD45^+^B202^+^), NK cells (CD45^+^NK1.1^+^) and neutrophils (CD45^+^Gr1^+^) (Fig. 2A, B). Then, the distribution of TAMs was investigated in tissues of breast cancer and paracarcinoma; we found that the expression level of PGRN was proportional to the expression level of CD68 and CD68^+^ macrophages were accumulated within and around the tumor tissues (Fig. 2C).

To make sure whether macrophages are involved in PGRN-mediated CD8^+^T cells exclusion, we have used an orthotopic model with transplantation of PY8119 cells into the mammary fat pad. As expected, in response to clodronate liposomes (CLs) [25, 26] treatment, the number of TAM was significantly reduced (Fig. 2D). We detected that the breast tumors of CL-treated mice containing higher infiltration of cytotoxic CD8^+^T cells grew slower (Fig. 2E, F). We also found that high fluorescence intensity was simultaneously captured by fluorescence microscope when the apoptosis occurred after treatment of CLs (Fig. 2G); however, analysis of the proliferation marker Ki67 indicated that CL-treated mice proportionally contained the same levels of Ki67 when compared with tumors from PBS mice (Fig. 2G). Together, these results suggested that the inhibition of TAMs by PGRN depletion promoted the infiltration and activation of CD8^+^T cell and impaired PGRN-mediated immunosuppressive effect.

### PGRN promoted immunosuppressive M2-like TAMs accumulation and induced CD8 + T cell exclusion

**Fig. 3:**
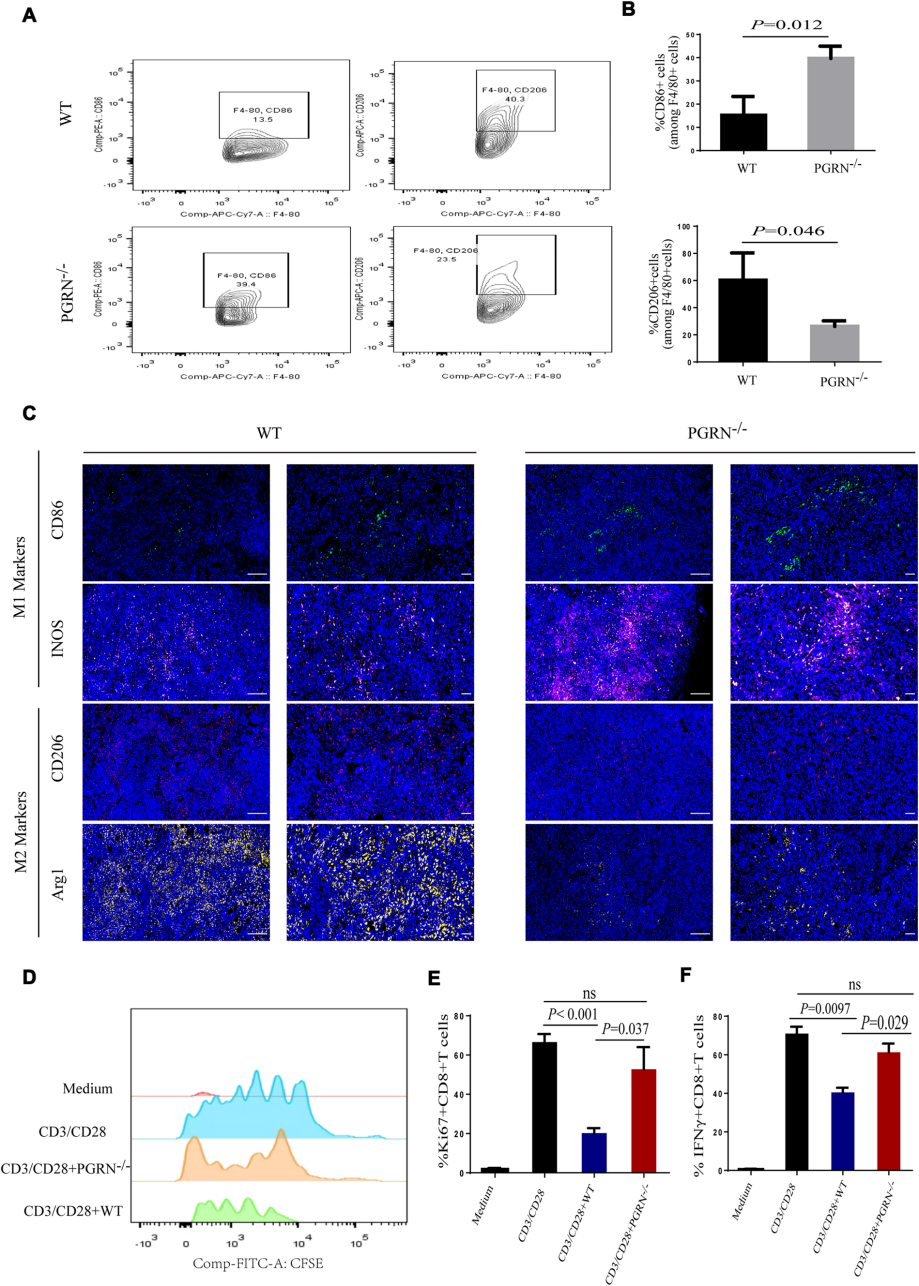
PGRN promoted immunosuppressive M2-like TAMs accumulate and induces CD8^+^T cells exclusion. **A**, **B** Percentage of M1 macrophages (F4/80^+^CD86^+^), M2 macrophages (F4/80^+^CD206^+^) overtime were assessed by flow cytometry analysis (**A**), quantification of M1 and M2 macrophages (**B**). **C** Immunofluorescent images of M1-like TAM markers and M2-like TAM markers; scale bar, 100um and 50um respectively. **D**–**F** TAMs isolated from breast cancer tissues were tested for their ability to suppress CD28/CD3 stimulated splenic CD8^+^T cell proliferation (CFSE dilution and Ki67.^+^ cells; **D**, **E**) and activation (IFN*γ* level; **F**)

Tumor-associated macrophages (TAMs) can suppress CD8^+^ T cell effector functions depending on their activation state (16, 34). To clarify the activation state of TAMs in tumor tissue, we analyzed expression of M1-like and M2-like TAM markers by flow cytometry. Interestingly, TAMs isolated from PGRN^−/−^ tumors exhibited higher expression of M1-like markers (CD86). In contrast, the expression of immunosuppressive M2-like markers was significantly enhanced in macrophages derived from WT tumor (mannose receptor C-type 1 Fig. 3A, B). Sections of PGRN^−/−^ tumor tissue showed a larger number of TAMs expressing pro-inflammatory markers, including iNOS, CD86 (Fig. 3C). On the contrary, PGRN WT tumors contained abundant M2-like macrophages expressing macrophage mannose receptor 1 (CD206) and arginase 1 (Arg1; Fig. 3C). As well known, M2-like macrophages were able to participate in potent inhibitory effects on cytotoxic CD8^+^T cell functions [41]. Comparing with TAMs isolated from PGRN^−/−^tumor tissue, we found TAMs isolated from WT group played significantly inhibitory role in CD8^+^T cell proliferation (Fig. 3D, E) and activation ex vivo (Fig. 3F). Together, our data indicated that PGRN promoted the polarization of TAMs toward M2-like cells that inhibited cytotoxic CD8^+^T cell functions.

### PGRN up-regulated ICAM-1 expression of TAMs mediating the interaction between TAMs with CD8 + T cells

**Fig. 4:**
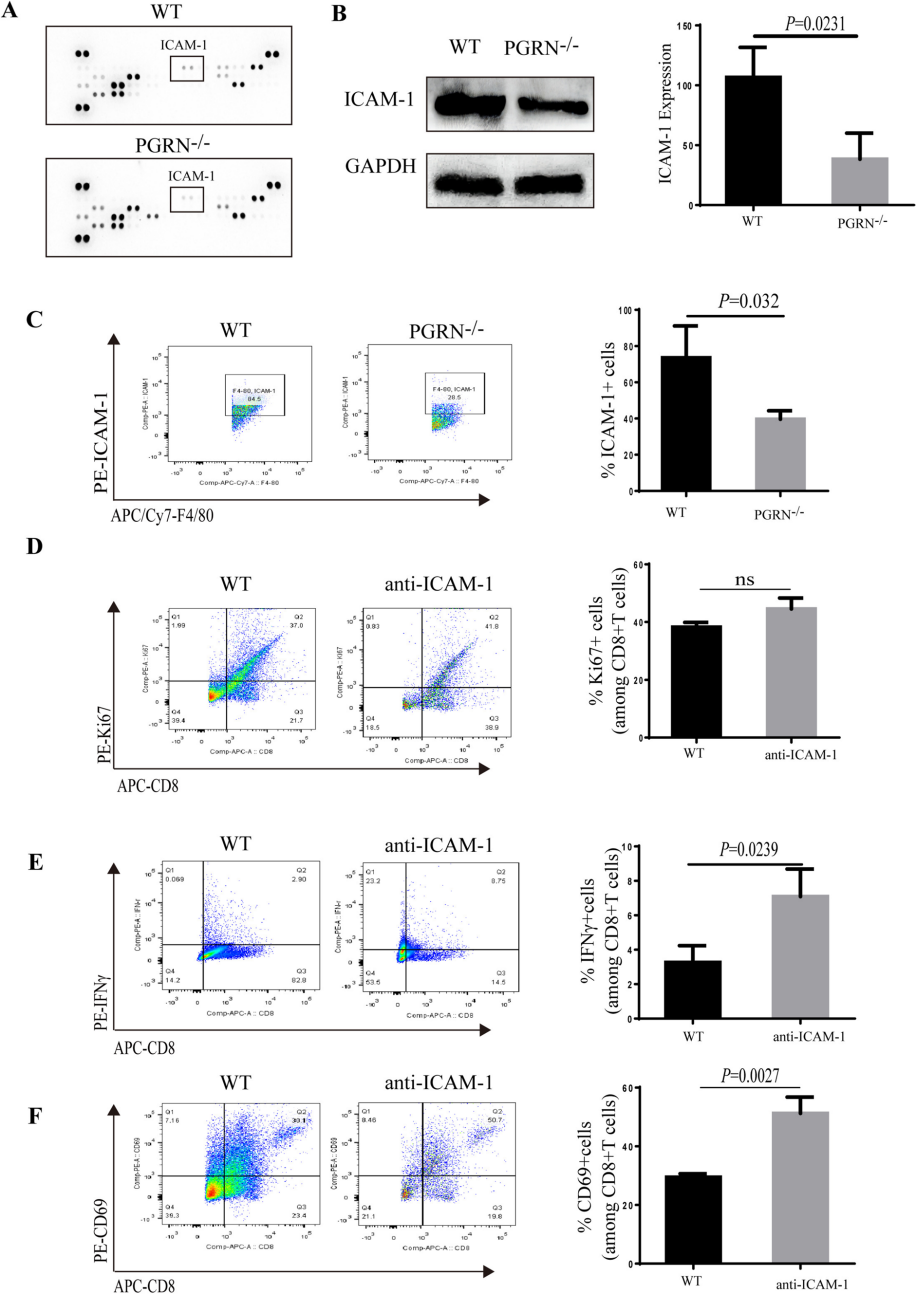
PGRN promoted CD8^+^T cells exclusion by up-regulating ICAM-1 of TAMs. **A** Mouse-specific chemokine protein array was performed with wild-type (WT) PMs CM and PGRN^−/−^ PMs CM. **B** Western blot was performed to analyze ICAM-1 expression and quantification. **C** Percentage of ICAM-1^+^ macrophages isolated from WT and PGRN^−/−^ BCa tissues was analyzed by flow cytometry and quantification. **D**–**F** TAMs isolated from WT tumor were tested for their ability to suppress CD28/CD3 stimulated splenic CD8^+^T cell proliferation (Ki67^+^ cells; **D**), activation (IFNγ; **E** and CD69 levels; **F**) with the presence or absence of ICAM-1 neutralizing antibody

We next identified the macrophage-derived factors responsible for CD8^+^ T cells exclusion. In this aspect, we exposed peritoneal macrophages of WT and PGRN^−/−^ groups to IL-4 in vitro, and then, we used cytokine array to examine secreted protein. In the WT group, among the most highly secreted proteins, we identified intercellular cell adhesion molecule-1 (ICAM-1, also known as CD54, Fig. 4A, B), a transmembrane glycoprotein in the immunoglobulin superfamily that has previously been shown to mediate T cell activation by interacting with the *β*2 integrin counter-receptors CD11a/CD18 (also known as lymphocyte function-associated antigen-1, LFA-1) [42]. Orthotopic models were used to isolate TAMs from WT and PGRN^−/−^ mice, by flow cytometry; we did find that ICAM-1 was highly expressed in the TAMs of WT murine tumor tissue (Fig. 4C). TAMs isolated from WT and PGRN^−/−^ mice were labeled with 5 mmol/L carboxyfluorescein diacetate succinimidyl ester (CFSE) and incubated with CD8 + T cells. The CFSE-labeled TAMs bound to T cells stimulated by anti-CD3/CD28 antibodies and TAMs derived from PGRN^−/−^ group showed a stronger capability of T cell binding than WT group, and the pre-treatment of TAMs with anti-ICAM-1 antibodies markedly decreased the binding of T cell and TAMs, which suggested the ICAM-1 was directly involved in the T cell-TAMs binding (Supplementary Figure S4).

Since our previous result has shown that TAMs from WT group played an inhibitory role on stimulated CD8 + T cells (Fig. 3D–F), we next examined the role of ICAM-1 in this inhibition. Interestingly, when spleen T cells were co-cultured with murine tumor-derived macrophages in the presence or absence of ICAM-1 neutralizing antibody, we found that there was no significant effect on the positive ratio of Ki67 in CD8^+^T cells, which indicated that ICAM-1 did not alter the proliferation of CD8^+^T cells (Fig. 4D). However, the presence of ICAM-1 neutralizing antibody significantly enhanced the positive ratios of IFN*γ* or CD69 (IFNγ: 2.9% to 8.75%; CD69: 30.1% to 50.7%), which suggested that the activation of CD8^+^T cell was efficiently restored by ICAM-1 neutralizing antibody (Fig. 4E, F). Thus, these data prompted that PGRN inhibited the antitumor effect of CD8^+^T cells dependent on the interaction of CD8^+^T cells with macrophages by ICAM-1.

### Pharmacologic blockade of the CSF-1/CSF-1R axis rewrote TAMs into M1-like phenotype and restored CD8 + T cell cytotoxicity in tumor-bearing mice

**Fig. 5:**
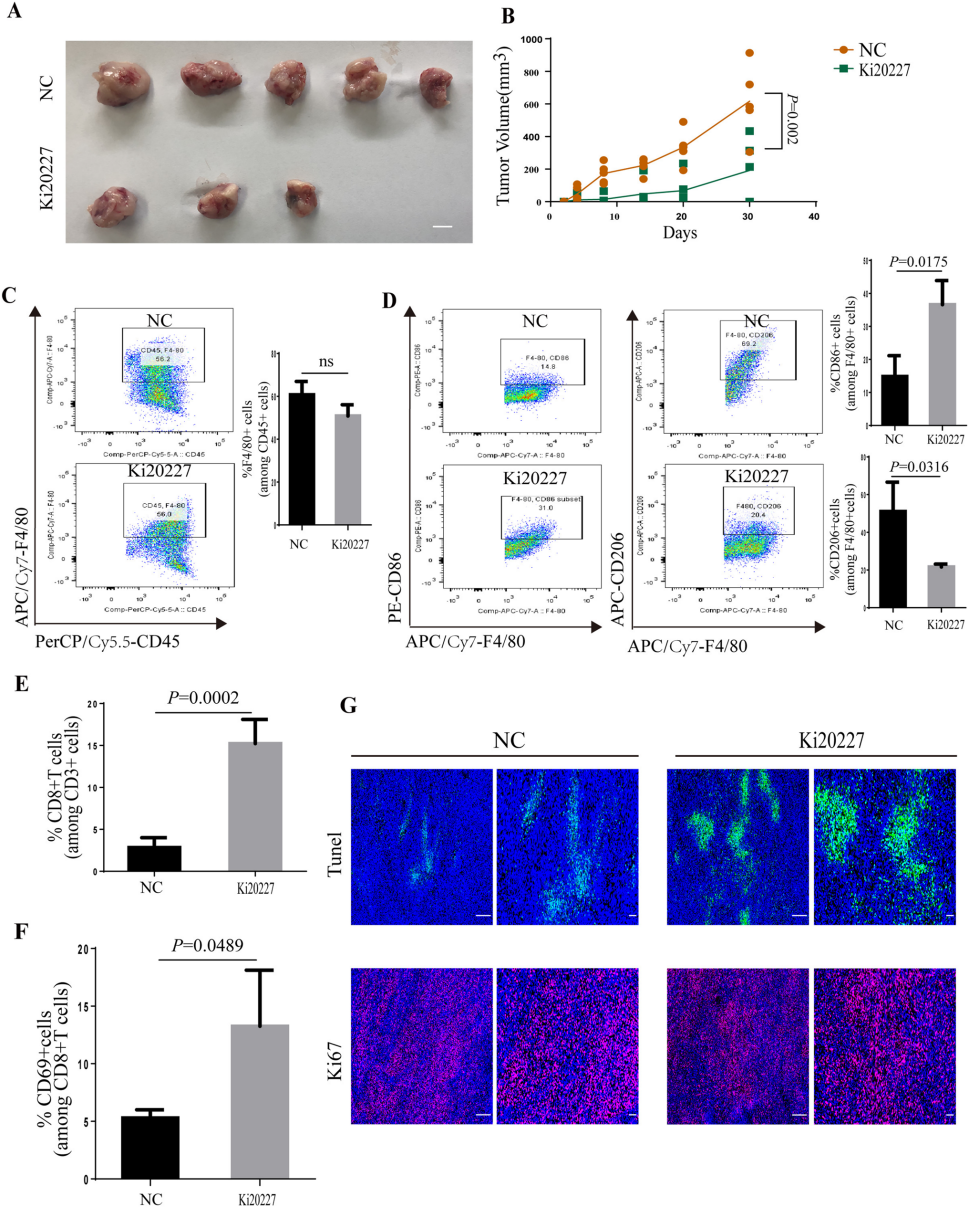
Pharmacologic blockade of the CSF-1/CSF-1R axis rewrote TAMs into M1-like phenotype and restores CD8^+^T cell cytotoxicity in tumor-bearing mice. **A**, **B** Breast cancer PY8119 cells were injected in situ into the fat pads of C57 wild-type mice (*n* = 5 per group) treated with a small molecule inhibitor of CSF-1R (Ki20227) (**A**); Tumor volume curve (**B**); scale bar,10mm. **C**, **D** Percentage of M1 macrophages (F4/80^+^CD86^+^), M2 macrophages (F4/80^+^CD206^+^) were assessed by flow cytometry analysis. **E**, **F** Quantification of CD3^+^CD8^+^T cells (**E**) and CD69^+^CD8^+^T cells (**F**) isolated from WT and Ki20227 tumor. **G** Representative immunofluorescent staining of Ki67^+^ and apoptotic cells (Tunel^+^) frequency in tumor treated with cosolvent and Ki20227; scale bar, 100um and 50um respectively

According to our findings, we reasoned that reprograming TAMs to M1-like TAMs could remain tumoricidal CD8^+^ T cell functions and subsequently suppress tumor progression. Targeting CSF-1/CSF-1R is one of the most well-known strategy to inhibit TAMs functions in cancer [24, 43]. Consequently, we investigated CSF-1R was largely expressed by TAMs (Supplementary Figure S2). To assess whether the blockade of CSF-1R affected macrophage and CD8^+^T cell functions, we used an orthotopic model with transplantation of PY8119 cells in the mammary fat pad. On day 7, tumor-bearing mice was treated with a small molecule inhibitor of CSF-1R (Ki20227) for 1 week. We found that the treatment of Ki20227 led to slower tumor growth and had a lower incidence of cancer (Fig. 5A, B). Interestingly, Ki20227 not only specifically reduced the number of M2-like F4/80^+^CD206^+^TAMs (Fig. 5C, D), but increased overall CD8^+^T cell numbers (Fig. 5E). Also, we found that CD8^+^T cell activation was increased in Ki20227-treated mice with a significant difference in Tunel^+^ cell numbers; meanwhile, analysis of the Ki67 showed that Ki20227-treated mice had no statistical difference compared with tumors from control mice (Fig. 5F, G). These findings demonstrated that the ability of TAMs to sustain tumor progression was partially due to their capacity to promote de-activation of CD8^+^ T cells, and TAM-targeted therapies induced the infiltration and activation of cytotoxic CD8^+^ T cells.

### Tumor-derived, not macrophage-derived PGRN regulated CD8 + T cell exclusion

**Fig. 6:**
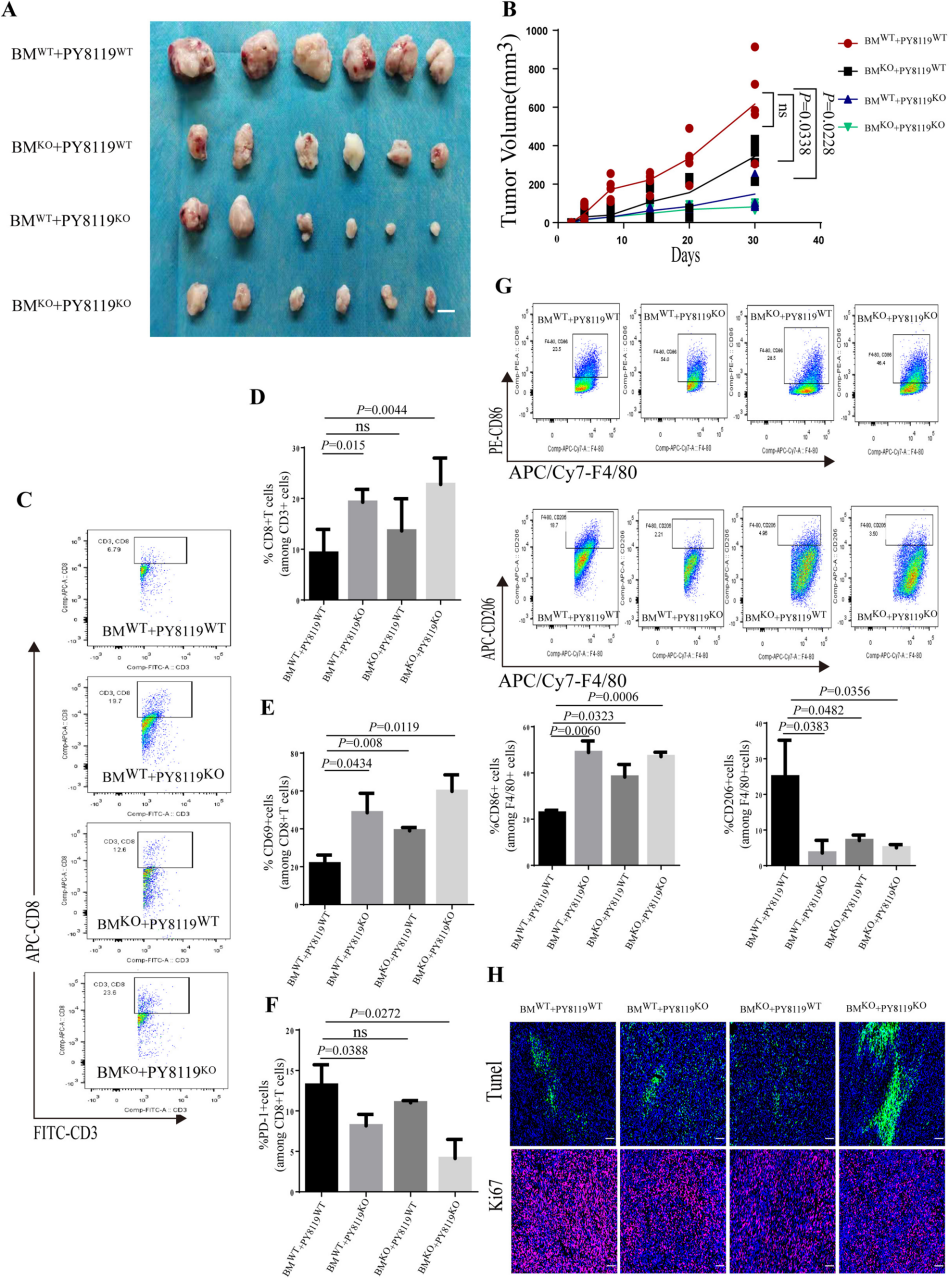
Tumor-derived, not macrophage-derived PGRN regulated CD8^+^ T cell exclusion. **A**, **B** Representative breast cancer PY8119 and PY8119/Grn^−/−^ cells were transplanted subcutaneously in right flank of the mice (*n* = 6 per group). Tumor tissues (**A**) and tumor volume curve (**B**); scale bar,10mm. **C**, **D** Percentage of CD3^+^CD8^+^T cells isolated from tumor was analyzed by flow cytometry (**C**) and quantification (**D**). **E**, **F** Quantification of CD8^+^T cell activation (CD69 and PD-1 levels). **G** Percentages of M1 macrophages (F4/80^+^CD86^+^) and M2 macrophages (F4/80^+^CD206^+^) were assessed by flow cytometry analysis and quantification. **H** Representative immunofluorescent staining of Ki67^+^ and apoptotic cells (Tunel^+^) frequency in tumor tissues; scale bar, 50um

To further address the question which cells PGRN was derived from for CD8^+^ T cell exclusion, either cancer cells or macrophages, we, respectively, generated monoclonal PY8119 cell line without PGRN (Grn^−/−^ PY8119^KO^), in which the endogenous Grn gene was deleted via CRISPR/Cas9 and chimeric Grn-deficient mice in the BM compartment (Grn^−/−^ BM^KO^ mice). In vitro, we investigated cell viability between PY8119^KO^ cells and PY8119^WT^ cells, there was no difference in the proliferation of PY8119^KO^ cells and PY8119^WT^ cells (Supplementary Figure S9). Then, we used a subcutaneous model with transplantation of murine breast tumor cells subcutaneously in right flank of the mice. Compared with WT PY8119, we figured out that Grn^−/−^PY8119 cells significantly lost the ability to grow; what is more, the breast tumor of chimeric mice (BM^KO^) injected with Grn^−/−^PY8119 cells (PY8119^KO^) grew slowest (Fig. 6A, B). Comparing with dual Grn WTgroup (BM^WT^ + PY8119^WT^), CD8^+^T cell numbers significantly increased in either dual Grn-/- group (BM^KO^ + PY8119^KO^, *P* = 0.0044) or Grn^−/−^PY8119 group (BM^WT^ + PY8119^KO^, *P* = 0.015), but not significantly in Grn^−/−^BM mice group (BM^KO^ + PY8119^WT^) (Fig. 6C, D). Moreover, depletion of PGRN altered CD69 and PD-1expression levels in CD8^+^T cells (Fig. 6E, F). Interestingly, we found the depletion of PGRN was able to increase the number of immune-stimulatory TAMs that expressed the pro-inflammatory marker CD86 in mice tumors (Fig. 6G), though the numbers of CD3^+^T cell, Gr-1^+^ neutrophil, NK1.1^+^NK cell and CD45R^+^B cell remained unchanged (Supplementary Figure S3A–D). Analysis of Ki67 suggested that the depletion of PGRN had no effect on tumor proliferation; however, in tumor tissue of dual Grn-/- group (BM^KO^ + PY8119^KO^), we found a high apoptosis rate (Tunel^+^) of tumor cells (Fig. 6H). Taken together, our data indicated that tumor-derived, not macrophage-derived PGRN, played a dominant role in CD8^+^T cell exclusion.

### Combining depletion of tumor-derived PGRN and the checkpoint inhibitor Anti–PD-1 increased CD8 + T cell recruitment at the tumor site and reduced tumor burden

**Fig. 7:**
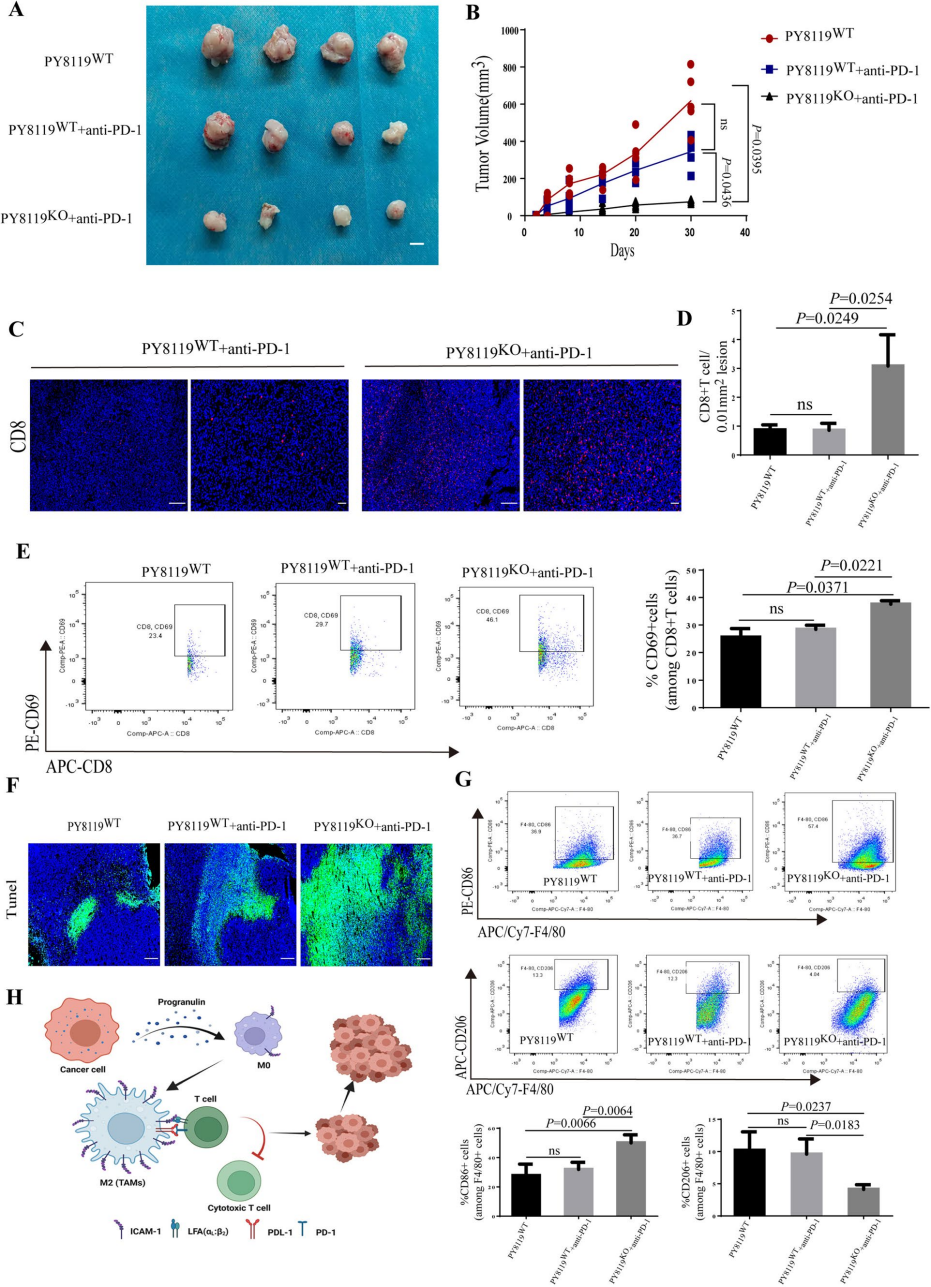
Combining depletion of tumor-derived PGRN and the checkpoint inhibitor anti–PD-1 increased CD8^+^T cell recruitment at the tumor site and reduced tumor burden. **A**, **B** Representative breast cancer PY8119 and PY8119/Grn^−/−^ cells were transplanted subcutaneously in right flank of the mice treated with anti-PD-1 therapy (*n* = 4 per group). Tumor tissues (**A**) and tumor volume curve (**B**); scale bar,10mm. Immunofluorescent images (**C**) and quantification (**D**) of CD8^+^T cells. **E** Flow cytometry analysis and quantification of CD8^+^T cell activation (CD69 level). **F** Immunofluorescent staining of apoptotic cells (Tunel^+^) frequency in tumor tissues. **G** Percentage of M1 macrophages (F4/80^+^CD86^+^), M2 macrophages (F4/80^+^CD206^+^) were assessed by flow cytometry analysis and quantification. **H** Schematic showing that tumor-derived progranulin mediates polarization of macrophages to suppress CD8^+^T cell infiltration. Tumor cells stimulate the polarization and recruitment of macrophages through progranulin secretion, result in excessive accumulation of M2 macrophages. Progranulin-induced TAMs express high level of the ICAM-1 and PD-L1, thereby enhancing TAMs to contact with CD8^+^ T cells and growth of breast cancer cells

Currently, pembrolizumab combined with chemotherapy did not well resolve the low response rate of anti-PD-1/PD-L1 therapy [44]. Since ablation of tumor-derived PGRN was accompanied by an increase of CD8^+^T cell number at the tumor site, we reasoned that next evaluated whether depletion of tumor-derived PGRN might modify efficacy of anti-PD-1 therapy. Mice with tumor were treated with the checkpoint inhibitor anti-PD-1 at day 7, for 2 weeks. In response to intervention, though anti-PD-1 alone was not able to reduce tumor burden, combination of anti-PD-1 treatment with depletion of tumor-derived PGRN led to a dramatic decrease of tumor burden (Fig. 7A, B) and a higher apoptosis rate (Fig. 7F). However, no difference in CD8^+^T cell recruitment was detected between PY8119 Grn WT tumors with treatment of anti-PD-1 (PY8119^WT^ + anti-PD-1) or without anti-PD-1 (PY8119^WT^);in contrast, CD8^+^T cell was obviously increased in PY8119/Grn^−/−^ tumors treated with anti-PD-1 ((PY8119^KO^ + anti-PD-1)(Fig. 7C, D). Moreover, anti-PD-1 treatment did not significantly alter CD69 expression levels in CD8^+^T cells isolated from PY8119 Grn WT tumors, whereas depletion of tumor-derived PGRN led to a significant up-regulation of CD69 expression in CD8^+^T cells in response to anti-PD-1 administration (Fig. 7E). Interestingly, we found that an increased presence of immune-stimulatory TAMs that expressed the pro-inflammatory marker CD86 in PY8119/Grn^−/−^ tumors treated with anti-PD-1. Reciprocally, these tumors displayed a lower rate of immunosuppressive CD206^+^TAMs compared with WT tumors or WT tumors treated with anti-PD-1 (Fig. 7G), These findings suggested that anti-PD-1 therapy had no effect on macrophage functions. Taken together, these data suggested that CD8^+^T cell infiltration was impeded by PGRN-induced CD8^+^T cells exhaustion which impaired the effectiveness of anti-PD-1 therapy, and restoration of CD8^+^T cell infiltration via PGRN depletion/inhibition was a prerequisite for effective anti-PD-1 therapy in the breast cancer.

## Discussion

This study shows that tumor-derived PGRN provides protection against the immune attack that induces of T cell exclusion in the primary breast cancer, and it is potentially used as preclinical data that support the feasibility for targeting tumor-derived PGRN combined with immune checkpoint blocker anti-PD-1 for the treatment of breast cancer and provide a new idea for the immunotherapy of breast cancer.

Immune evasion plays an important role in the occurrence and evolution of cancer, mainly allowing malignant tumor cells to escape the surveillance of the body’s immune system and avoid being destroyed by effector cells [1]. PGRN as a growth factor is critically involved in a wide variety of physiological and pathological processes. Recently, numerous studies have pointed out its importance in tumor cell proliferation [32, 33, 35], migration, invasion [45, 46], angiogenesis [47], and anti-inflammatory, as our previous work revealed that MiR-5100 of PGRN^−/−^ TAMs derived exosomes inhibited the CXCL12/CXCR4 axis and then ultimately inhibited the invasion, migration and EMT of breast cancer cells [48]; however, its role in immune evasion of breast cancer is poorly understood. Our date demonstrated that CD8^+^T cell function and infiltration being lost in human and mouse breast cancer tumor with characteristically a lot of PGRN; moreover, the PGRN is mainly derived from tumor cells.

Macrophages have important functions in immune responses and adopt various phenotypes, mainly depending on the environment including the secretion of tumor-derived mediators, hypoxic and necrotic factors [49, 50]. When we depleted TAMs by clodronate liposomes or a small molecule inhibitor of CSF-1R (Ki20227), we found that PGRN-mediated immunosuppressive effect was impaired. Previous studies suggested that immunosuppressive cytokines released by M2 macrophages inhibited the function and metabolism of T cells [43, 51]. Thus, it may be interesting and meaningful for next research to determine which receptor plays a major role in PGRN-mediated breast cancer immunosuppression. When we insolated TAMs from WT mice, we found that their ICAM-1 protein levels were increased. ICAM-1 is a member of the immunoglobulin superfamily of proteins expressed in all leukocytes and on the surface of many cancer cell types, which shows altered expression in malignant diseases and involved in the process of cancer metastasis [52]. Our previous work found that the PGRN inhibited the proliferation and activation of CD8^+^T cells through the interaction of PD-L1 on TAMs with PD-1 on CD8^+^T cells [37]. However, blocking the interaction of PD-L1 and PD-1by PD-1 antibody did not completely abolish the suppressing effect on CD8^+^T cells, which indicated that there were other mechanisms in addition to it. ICAM-1 binds to ligands with a high affinity, providing a physical force for the adhesion between cells prolonging interaction time between cells and strengthening affinities between proteins. In our work, in vitro experiments have demonstrated that antibody blocking of ICAM-1 reduced the interaction between TAMs and CD8 + T cells and attenuated the suppressive effect of TAMs on CD8 + T cell activation, whereas attenuated the affinities between PD-L1 and PD-1.

Dependent on the microenvironment, on the one hand, macrophages are able to promote tumor growth; on the other hand, they critically play an antitumor immune response [41]. Therapies that aim to specifically inhibit the immunosuppressive functions of macrophages, while sparing and/or enhancing their antitumor immune response, could act as an alternative and, perhaps, more efficient approach than therapies that reduce macrophage numbers in tumors [53, 54]. In this regard, our studies indicate that depletion of tumor-derived PGRN is sufficient therapies to restore T cell infiltration and function at the breast cancer and characteristically surrounded by low numbers of M2-like macrophages. Thus, PGRN deficient in tumor cell can access an abundant number of macrophages for their reprogramming toward an immune-stimulatory M1-like phenotype, which facilitates the mounting of an effective immune response against cancer.

Central to the efficacy of immune checkpoint blockade is the requirement for cytotoxic CD8^+^ T cells to infiltrate into tumors [1, 17]. Since TGF-*β* promoted the formation of immune-excluded tumors by increasing peritumoral collagen production of cancer-associated fibroblast(CAF), recently, YM101 and BiTP were developed to address this issue, which were developed based on the symmetric tetravalency BsAb technology. However, in vivo experiments showed that BiTP significantly retarded tumor growth and prolonged survival of tumor-bearing mice; in clinical trial data, YM101 and BiTP were not used in the clinical trial [55]. Considering the unsatisfactory response rate of anti-PD-1/PD-L1, our previous work found that the PGRN promoted the dysfunction of CD8 + T cells through the interaction of PD-L1 on TAMs with PD-1 on CD8^+^T cells [53], these work give us a reasonable idea: Whether the combination of ablation of PGRN and anti-PD-1 therapy showed better antitumor effects in breast cancer?

Indeed, in this study, we find that breast tumor becomes more sensible to anti-PD-1 therapy by combining PGRN depletion accompanied by increased numbers of M1-like macrophages. In conclusion, our study reveals a mechanism by which breast tumor evades the CD8^+^ T cell immune response and provides the rationale for potential therapy by targeting PGRN in combination with immune checkpoint inhibitors for breast cancer patients.

### Supplementary Information

Below is the link to the electronic supplementary material.

Supplementary file1 (DOCX 31352 kb)

## Abbreviations

Anti-PD-1: Anti-PD-1
BCa: Breast cancer
CFSE: Carboxyfluorescein diacetate succinimidyl ester
CL: Clodronate liposome
CTL: Cytotoxic T cell
CSF-1: Colony-stimulating factor-1
CSF-1R: Colony-stimulating factor-1 receptor
ICAM-1: Intercellular cell adhesion molecule-1
NK cell: Natural killer cell
PD-1: Programmed cell death protein 1
PGRN: Progranulin
TAM: Tumor-associated macrophage
TME: Tumor micro-environment

## References

[1] Chen DS, Mellman I (2013). Oncology meets immunology: the cancer-immunity cycle. Immunity.

[2] Fridman WH, Pagès F, Sautès-Fridman C, Galon J (2012). The immune contexture in human tumours: impact on clinical outcome. Nat Rev Cancer.

[3] Fukunaga A, Miyamoto M, Cho Y, Murakami S, Kawarada Y, Oshikiri T, Kato K, Kurokawa T, Suzuoki M, Nakakubo Y (2004). CD8+ tumor-infiltrating lymphocytes together with CD4+ tumor-infiltrating lymphocytes and dendritic cells improve the prognosis of patients with pancreatic adenocarcinoma. Pancreas.

[4] Gupta R, Bhatt LK, Johnston TP, Prabhavalkar KS (2019). Colon cancer stem cells: potential target for the treatment of colorectal cancer. Cancer Biol Ther.

[5] Oshi M, Asaoka M, Tokumaru Y, Li Y, Matsuyama R, Ishikawa T, Endo I, Takabe K (2020). CD8 T cell score as a prognostic biomarker for triple negative breast cancer. Int J Mol Sci.

[6] Dangaj D, Bruand M, Grimm AJ, Ronet C, Barras D, Duttagupta PA, Lanitis E, Duraiswamy J, Tan Yi JL, Benencia F (2019). Cooperation between constitutive and inducible chemokines enables T cell engraftment and immune attack in solid tumors. Cancer Cell.

[7] Topalian S, Drake C, Pardoll D (2015). Immune checkpoint blockade: a common denominator approach to cancer therapy. Cancer Cell.

[8] Conklin MW, Keely PJ (2012). Why the stroma matters in breast cancer: insights into breast cancer patient outcomes through the examination of stromal biomarkers. Cell Adh Migr.

[9] Brahmer JR, Tykodi SS, Chow L, Hwu WJ, Topalian SL, Hwu P, Drake CG, Camacho LH, Kauh J, Odunsi K (2012). Safety and activity of anti-PD-L1 antibody in patients with advanced cancer. J Urol.

[10] Mediratta K, El-Sahli S, D’Costa V, Wang L (2020). Current progresses and challenges of immunotherapy in triple-negative breast cancer. Cancers.

[11] Jalalvand M, Darbeheshti F, Rezaei N (2021). Immune checkpoint inhibitors: review of the existing evidence and challenges in breast cancer. Immunotherapy.

[12] Hong Y, Kim N, Li C, Jeong E, Yoon S (2017). Patient sample-oriented analysis of gene expression highlights extracellular signatures in breast cancer progression. Biochem Biophys Res Commun.

[13] Parker MW, Rossi D, Peterson M, Smith K, Bitterman PB (2014). Fibrotic extracellular matrix activates a profibrotic positive feedback loop. J Clin Investig.

[14] Won KA, Spruck C (2020). Triple-negative breast cancer therapy: current and future perspectives. Int J Oncol.

[15] Shree T, Olson OC, Elie BT, Kester JC, Garfall AL, Simpson K, Bell-McGuinn KM, Zabor EC, Brogi E, Joyce JA (2011). Macrophages and cathepsin proteases blunt chemotherapeutic response in breast cancer. Genes Dev.

[16] Hua W, Najibi AJ, Sobral MC, Bo RS, Mooney DJ (2020). Biomaterial-based scaffold for in situ chemo-immunotherapy to treat poorly immunogenic tumors. Nat Commun.

[17] Joyce JA, Fearon DT (2015). T cell exclusion, immune privilege, and the tumor microenvironment. Science.

[18] Gajewski TF, Schreiber H, Fu Y-X (2013). Innate and adaptive immune cells in the tumor microenvironment. Nat Immunol.

[19] Biswas SK, Allavena P, Mantovani A (2013) Tumor-associated macrophages: functional diversity, clinical significance, and open questions. In: Seminars in immunopathology, Springer, Berlin, pp 585–600 10.1007/s00281-013-0367-7 23657835

[20] Noy R, Pollard JW (2014). Tumor-associated macrophages: from mechanisms to therapy. Immunity.

[21] Zhao X, Qu J, Sun Y, Wang J, Liu X, Wang F, Zhang H, Wang W, Ma X, Gao X (2017). Prognostic significance of tumor-associated macrophages in breast cancer: a meta-analysis of the literature. Oncotarget.

[22] Komohara Y, Fujiwara Y, Ohnishi K, Takeya M (2016). Tumor-associated macrophages: potential therapeutic targets for anti-cancer therapy. Adv Drug Deliv Rev.

[23] Li J, Wang S, Wang N, Zheng Y, Yang B, Wang X, Zhang J, Pan B, Wang Z (2021). Aiduqing formula inhibits breast cancer metastasis by suppressing TAM/CXCL1-induced Treg differentiation and infiltration. Cell Communi Signal.

[24] Ries CH, Cannarile MA, Hoves S, Benz J, Wartha K, Runza V, Rey-Giraud F, Pradel LP, Feuerhake F, Klaman I (2014). Targeting tumor-associated macrophages with anti-CSF-1R antibody reveals a strategy for cancer therapy. Cancer Cell.

[25] Zhu Y, Knolhoff BL, Meyer MA, Nywening TM, West BL, Luo J, Wang-Gillam A, Goedegebuure SP, Linehan DC, DeNardo DG (2014). CSF1/CSF1R blockade reprograms tumor-infiltrating macrophages and improves response to T-cell checkpoint immunotherapy in pancreatic cancer models. Cancer Res.

[26] Strachan DC, Ruffell B, Oei Y, Bissell MJ, Coussens LM, Pryer N, Daniel D (2013). CSF1R inhibition delays cervical and mammary tumor growth in murine models by attenuating the turnover of tumor-associated macrophages and enhancing infiltration by CD8+ T cells. Oncoimmunology.

[27] Mitchem JB, Brennan DJ, Knolhoff BL, Belt BA, Zhu Y, Sanford DE, Belaygorod L, Carpenter D, Collins L, Piwnica-Worms D (2013). Targeting tumor-infiltrating macrophages decreases tumor-initiating cells, relieves immunosuppression, and improves chemotherapeutic responses. Cancer Res.

[28] DeNardo DG, Brennan DJ, Rexhepaj E, Ruffell B, Shiao SL, Madden SF, Gallagher WM, Wadhwani N, Keil SD, Junaid SA (2011). Leukocyte complexity predicts breast cancer survival and functionally regulates response to chemotherapy. Cancer Discov.

[29] Almeida S, Zhou L, Gao F-B (2011). Progranulin, a glycoprotein deficient in frontotemporal dementia, is a novel substrate of several protein disulfide isomerase family proteins. PLoS ONE.

[30] Yan W, Ding A, Kim H-J, Zheng H, Wei F, Ma X (2016). Progranulin controls sepsis via C/EBPα-regulated Il10 transcription and ubiquitin ligase/proteasome-mediated protein degradation. J Immunol.

[31] Feng T, Zheng L, Liu F, Xu X, Mao S, Wang X, Liu J, Lu Y, Zhao W, Yu X (2016). Growth factor progranulin promotes tumorigenesis of cervical cancer via PI3K/Akt/mTOR signaling pathway. Oncotarget.

[32] He Z, Bateman A (2003). Progranulin (granulin-epithelin precursor, PC-cell-derived growth factor, acrogranin) mediates tissue repair and tumorigenesis. J Mol Med.

[33] Kong W-J, Zhang S-L, Chen X, Zhang S, Wang Y-J, Zhang D, Sun Y (2007). PC cell-derived growth factor overexpression promotes proliferation and survival of laryngeal carcinoma. Anticancer Drugs.

[34] Bandey I, Chiou S, Huang A, Tsai J, Tu P (2015). Progranulin promotes temozolomide resistance of glioblastoma by orchestrating DNA repair and tumor stemness. Oncogene.

[35] Wang W, Hayashi J, Kim WE, Serrero G (2003). PC cell-derived growth factor (granulin precursor) expression and action in human multiple myeloma. Clin Cancer Res.

[36] Purrahman D, Mahmoudian-Sani M-R, Saki N, Wojdasiewicz P, Kurkowska-Jastrzębska I, Poniatowski ŁA (2022). Involvement of progranulin (PGRN) in the pathogenesis and prognosis of breast cancer. Cytokine.

[37] Fang W, Zhou T, Shi H, Yao M, Zhang D, Qian H, Zeng Q, Wang Y, Jin F, Chai C (2020) Progranulin induces immune escape in breast cancer via up-regulating PD-L1 expression on TAMS and promoting CD8+T cell exclusion 10.1186/s13046-020-01786-6 PMC7780622 33390170

[38] Zhang X, Goncalves R, Mosser DM (2008). The isolation and characterization of murine macrophages. Curr Protoc Immunol.

[39] Stylianopoulos T, Munn LL, Jain RK (2018). Reengineering the physical microenvironment of tumors to improve drug delivery and efficacy: from mathematical modeling to bench to bedside. Trends Cancer.

[40] Choi I-K, Strauss R, Richter M, Yun C-O, Lieber A (2013). Strategies to increase drug penetration in solid tumors. Front Oncol.

[41] Kitamura T, Qian B-Z, Pollard JW (2015). Immune cell promotion of metastasis. Nat Rev Immunol.

[42] Wingren AG, Parra E, Varga M, Kalland T, Sjogren H-O, Hedlund G, Dohlsten M (2017) T cell activation pathways: B7, LFA-3, and ICAM-1 shape unique T cell profiles. Crit Rev™ Immunol 37 10.1615/CritRevImmunol.v37.i2-6.130 29773030

[43] Mantovani A, Marchesi F, Malesci A, Laghi L, Allavena P (2017). Tumour-associated macrophages as treatment targets in oncology. Nat Rev Clin Oncol.

[44] Schmid P, Cortes J, Dent R, Pusztai L, McArthur H, Kümmel S, Bergh J, Denkert C, Park YH, Hui R (2022). Event-free survival with pembrolizumab in early triple-negative breast cancer. N Engl J Med.

[45] Liu Y, Xi L, Liao G, Wang W, Tian X, Wang B, Chen G, Han Z, Wu M, Wang S (2007). Inhibition of PC cell-derived growth factor (PCDGF)/granulin-epithelin precursor (GEP) decreased cell proliferation and invasion through downregulation of cyclin D and CDK 4 and inactivation of MMP-2. BMC Cancer.

[46] Tangkeangsirisin W, Serrero G (2004). PC cell-derived growth factor (PCDGF/GP88, progranulin) stimulates migration, invasiveness and VEGF expression in breast cancer cells. Carcinogenesis.

[47] Eguchi R, Nakano T, Wakabayashi I (2017). Progranulin and granulin-like protein as novel VEGF-independent angiogenic factors derived from human mesothelioma cells. Oncogene.

[48] Yue S, Ye X, Zhou T, Gan D, Qian H, Fang W, Yao M, Zhang D, Shi H, Chen T (2021). PGRN−/− TAMs-derived exosomes inhibit breast cancer cell invasion and migration and its mechanism exploration. Life Sci.

[49] Biswas SK, Mantovani A (2010). Macrophage plasticity and interaction with lymphocyte subsets: cancer as a paradigm. Nat Immunol.

[50] Ruffell B, Coussens LM (2015). Macrophages and therapeutic resistance in cancer. Cancer Cell.

[51] Osipov A, Saung MT, Zheng L, Murphy AG (2019). Small molecule immunomodulation: the tumor microenvironment and overcoming immune escape. J Immunother Cancer.

[52] Hayes SH, Seigel GM (2009). Immunoreactivity of ICAM-1 in human tumors, metastases and normal tissues. Int J Clin Exp Pathol.

[53] Bronte V, Murray PJ (2015). Understanding local macrophage phenotypes in disease: modulating macrophage function to treat cancer. Nat Med.

[54] Quail DF, Joyce JA (2013). Microenvironmental regulation of tumor progression and metastasis. Nat Med.

[55] Yi M, Wu Y, Niu M, Zhu S, Zhang J, Yan Y, Zhou P, Dai Z, Wu K (2022) Anti-TGF-β/PD-L1 bispecific antibody promotes T cell infiltration and exhibits enhanced antitumor activity in triple-negative breast cancer. J Immunother Cancer 10 10.1136/jitc-2022-005543 PMC9723957 36460337

