# Supplementary material for: PGRN inhibits CD8+T cell recruitment and promotes breast cancer progression by up-regulating ICAM-1 on TAM
Source: Cancer Immunol Immunother. 2024 Mar 30;73(5):76. doi: 10.1007/s00262-024-03655-z (PMC10981592; doi:10.1007/s00262-024-03655-z)
Supplement: Supplementary file 1 — Supplementary file1 (DOCX 31352 kb) [file 262_2024_3655_MOESM1_ESM.docx]

**Supplementary Material**


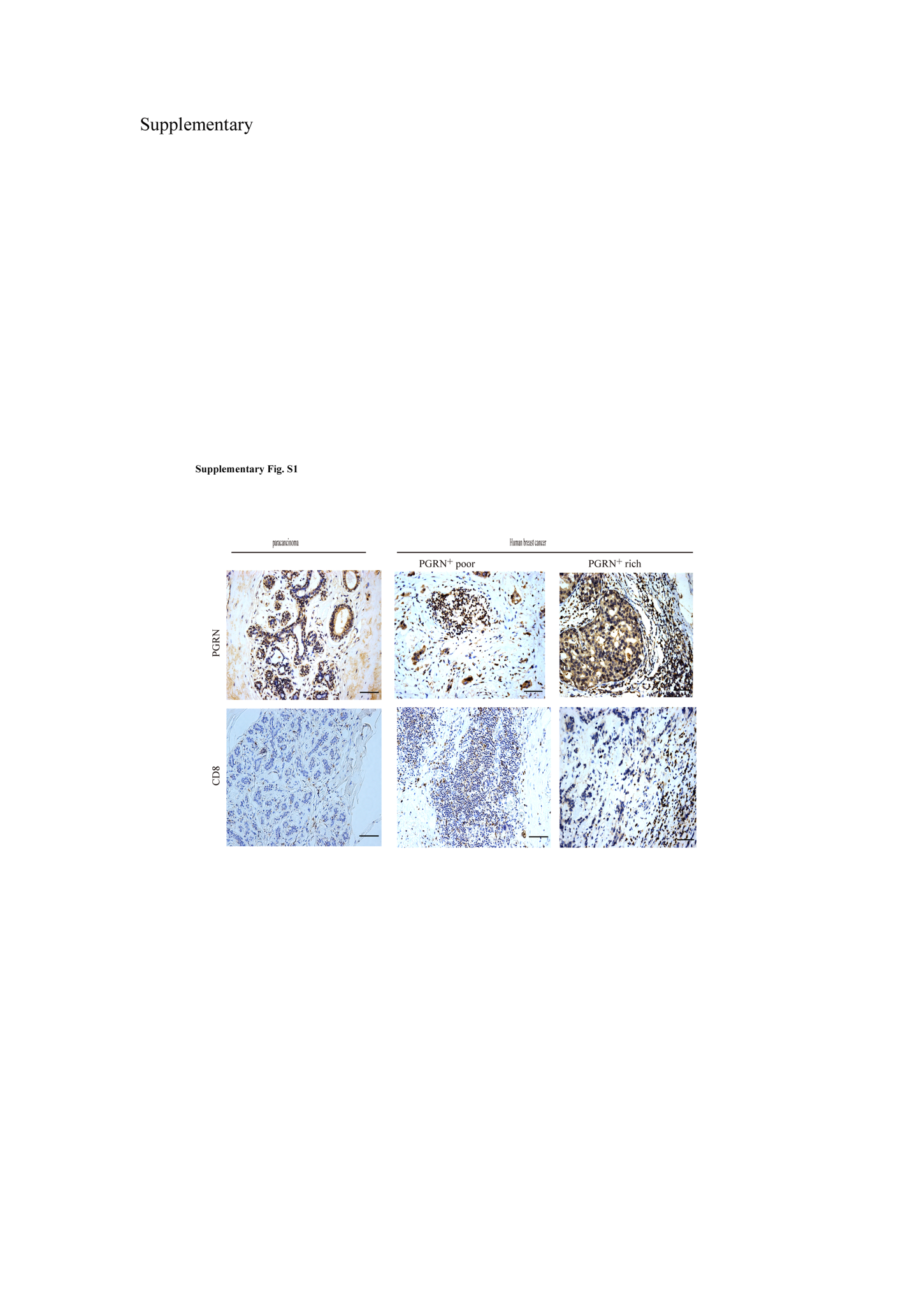


**Supplementary Figure 1. PGRN suppressed CD8^+^T cell infiltration** **in breast cancer.**

IHC images of PGRN^+^cells and CD8^+^T cells in human breast cancer and paracancerous tissue sections (n=68).


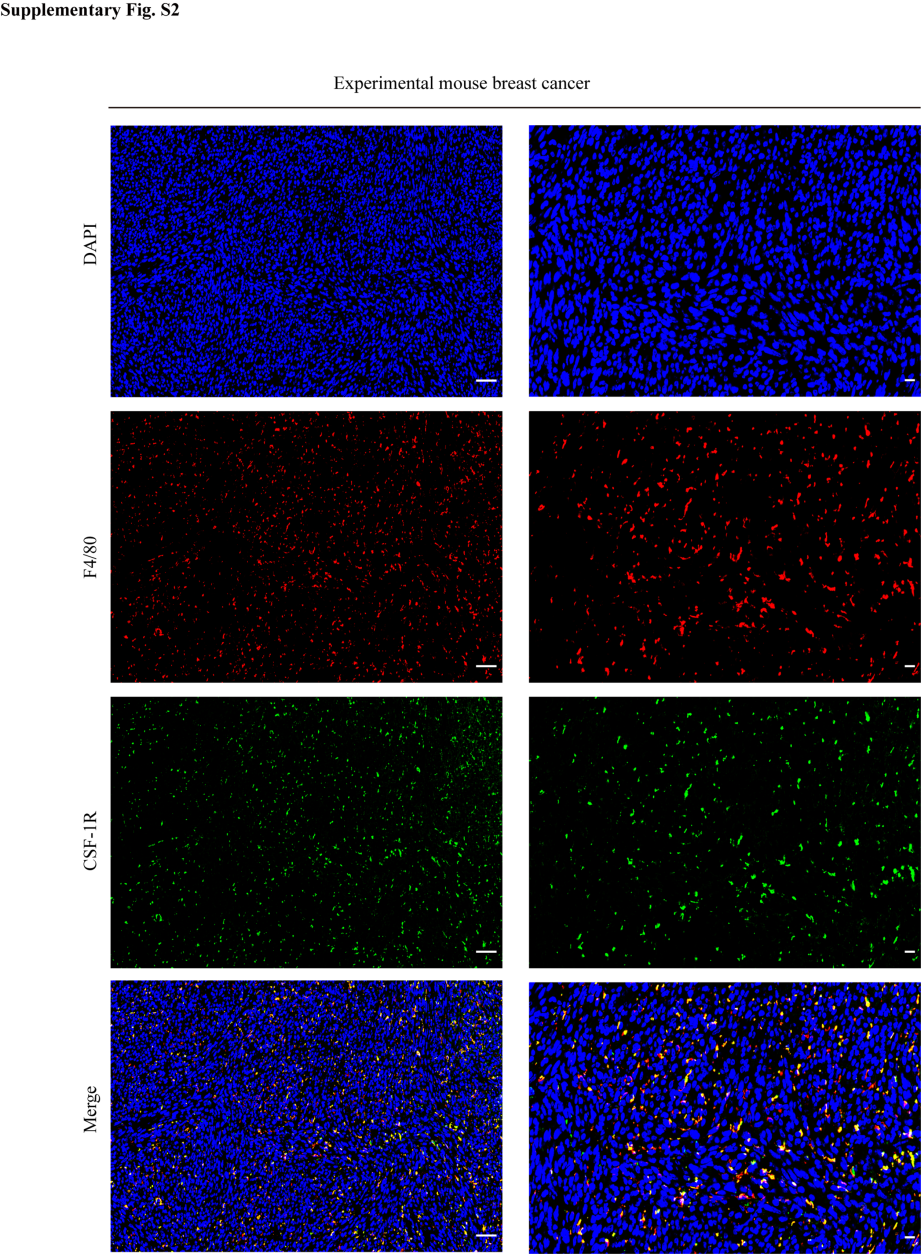


**Supplementary Figure 2. Macrophages expressed high level of the CSF1R in breast cancer.**

Representative images of MxIHC staining in tumors from WT and PGRN^-/-^mice; scale bar, 50um and 20um respectively.

**
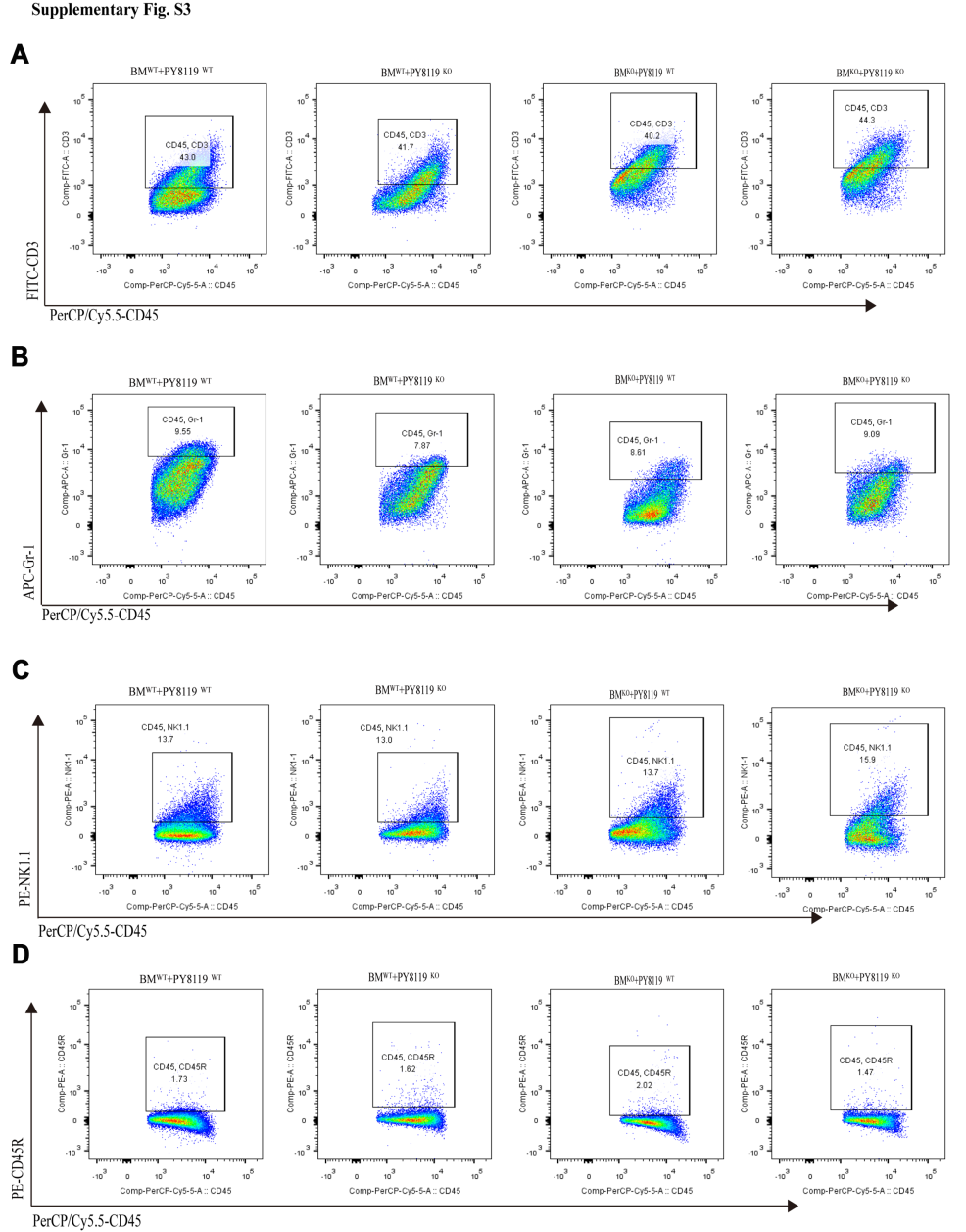
**

**Supplementary Figure 3. The number of B cells, T cells, NK cells and neutrophils remained unchanged in the knockout of tumor-derived progranulin.**

Composition of leukocytes is shown as a percentage of CD45^+^ cells using the following definitions: (A) T cells (CD45^+^CD3^+^), (B) neutrophils (CD45^+^Gr-1^+^) , (C) NK cells (CD45^+^NK1.1^+^) and (D) B cells (CD45^+^CD45R^+^) by flow cytometry analysis.


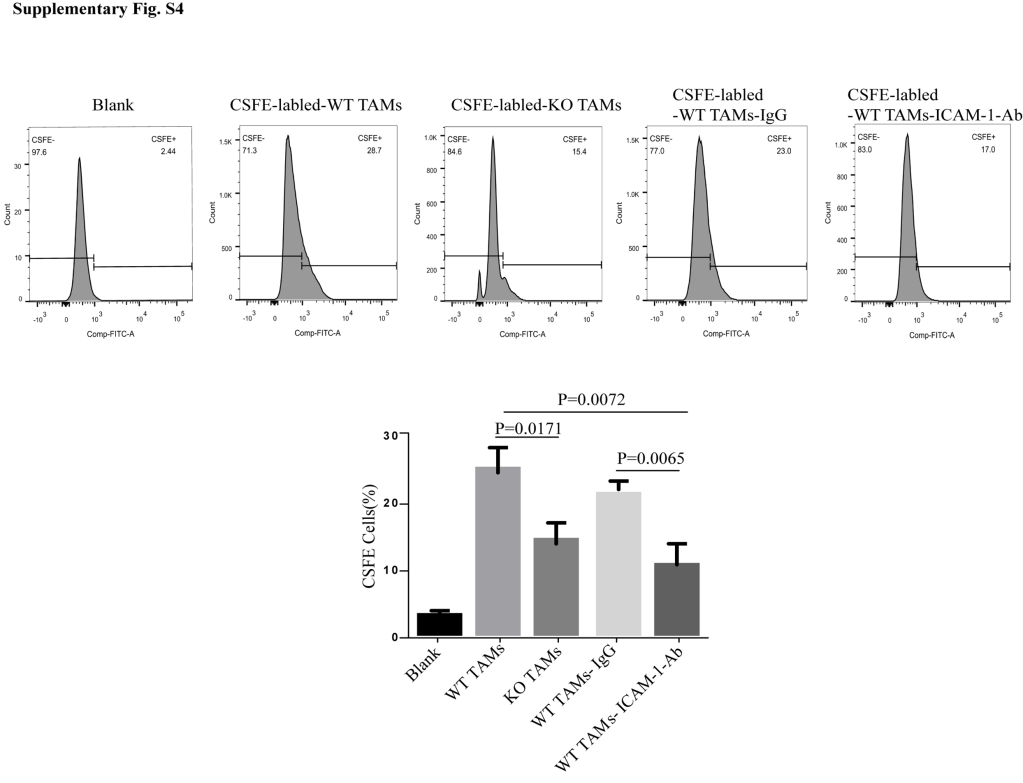


**Supplementary Figure 4. ICAM-1 mediates the adhesion between TAMs and CD8^+^ T lymphocytes.**

Representative histograms of CD8+ T cells bound to CFSE TAMs that were pre-treated with IgG or anti-ICAM-1 antibodies or derived from WT or PGRN^-/-^ mice. The proportions of CFSE-positive CD8+ T cells are shown on the right.


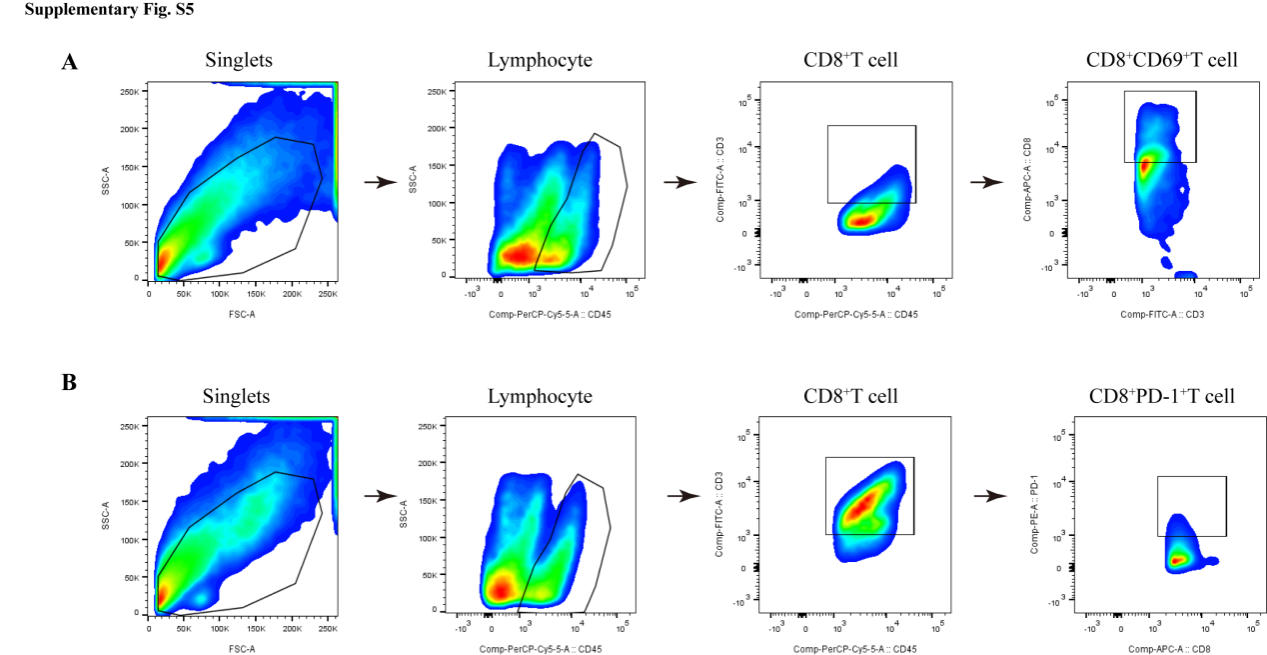


**Supplementary Figure 5.** The gating strategies for CD8^+^ CD69^+^T cell (A) and CD8^+^ PD-1^+^T cell （B）in flow cytometry assays.


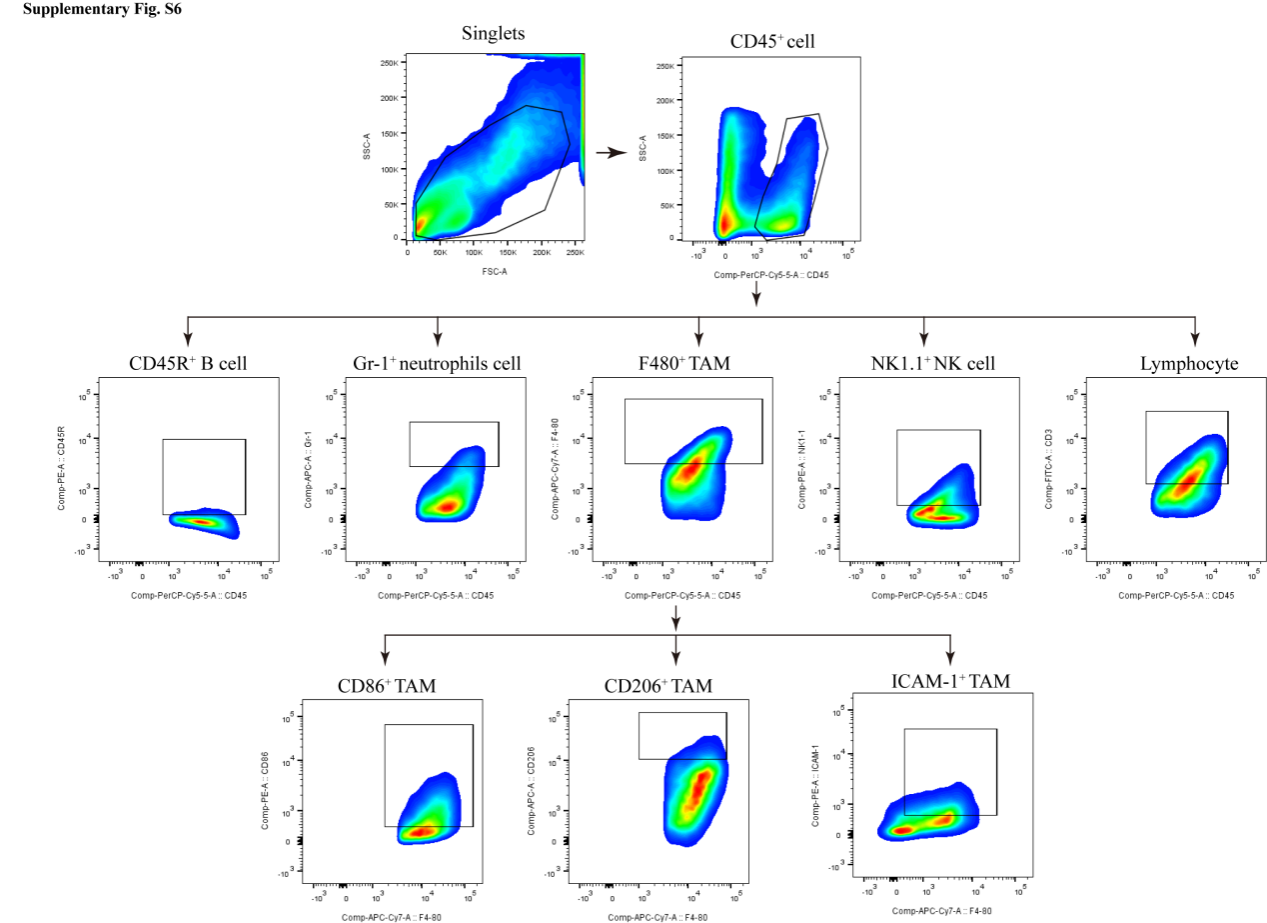


**Supplementary Figure 6.** The gating strategies for B cells (CD45^+^CD45R^+^), T cells (CD45^+^CD3^+^), NK cells (CD45^+^NK1.1^+^), neutrophils (CD45^+^Gr-1^+^), TAMs (CD45^+^F4/80^+^), M1 macrophages (F4/80^+^CD86^+^) , M2 macrophages (F4/80^+^CD206^+^) and ICAM-1^+^ TAMs in flow cytometry assays.


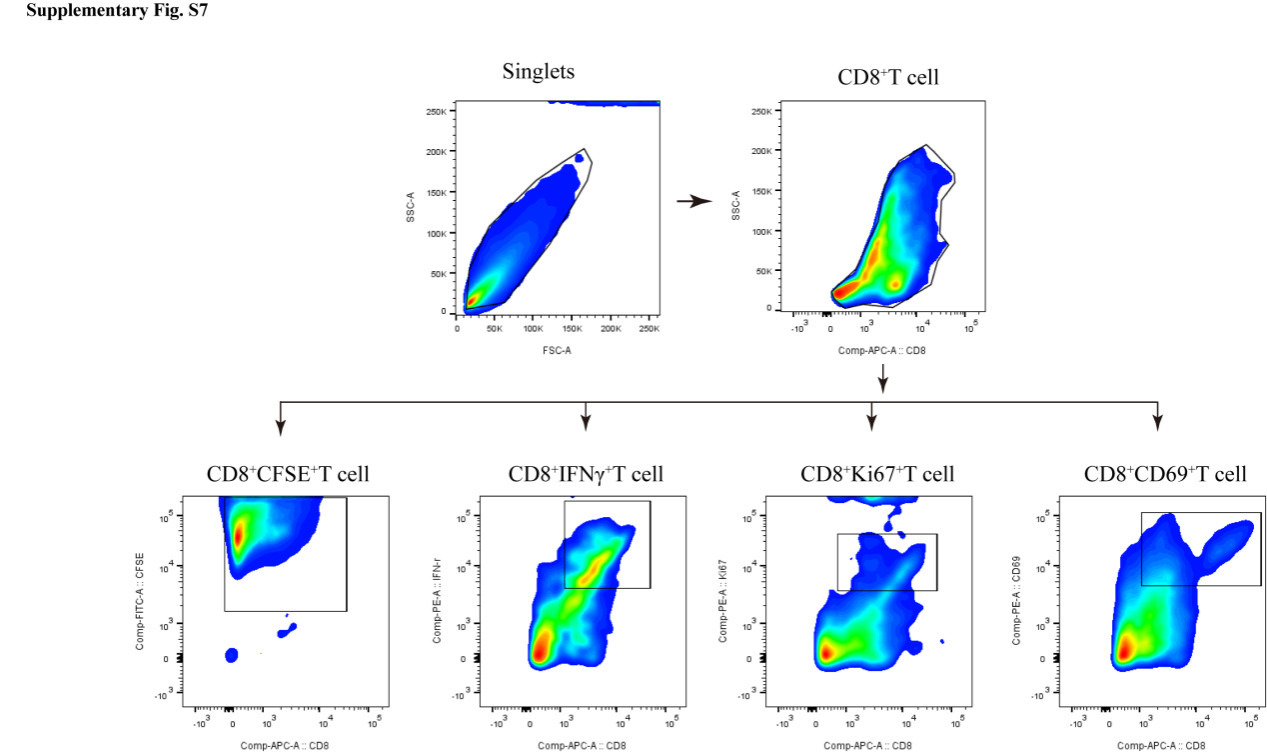


**Supplementary Figure 7.** The gating strategies for CD8^+^CFSE^+^T cell ,CD8^+^IFNγ^+^T cell, CD8^+^Ki67^+^T cell and CD8^+^ CD69^+^T cell in flow cytometry assays.


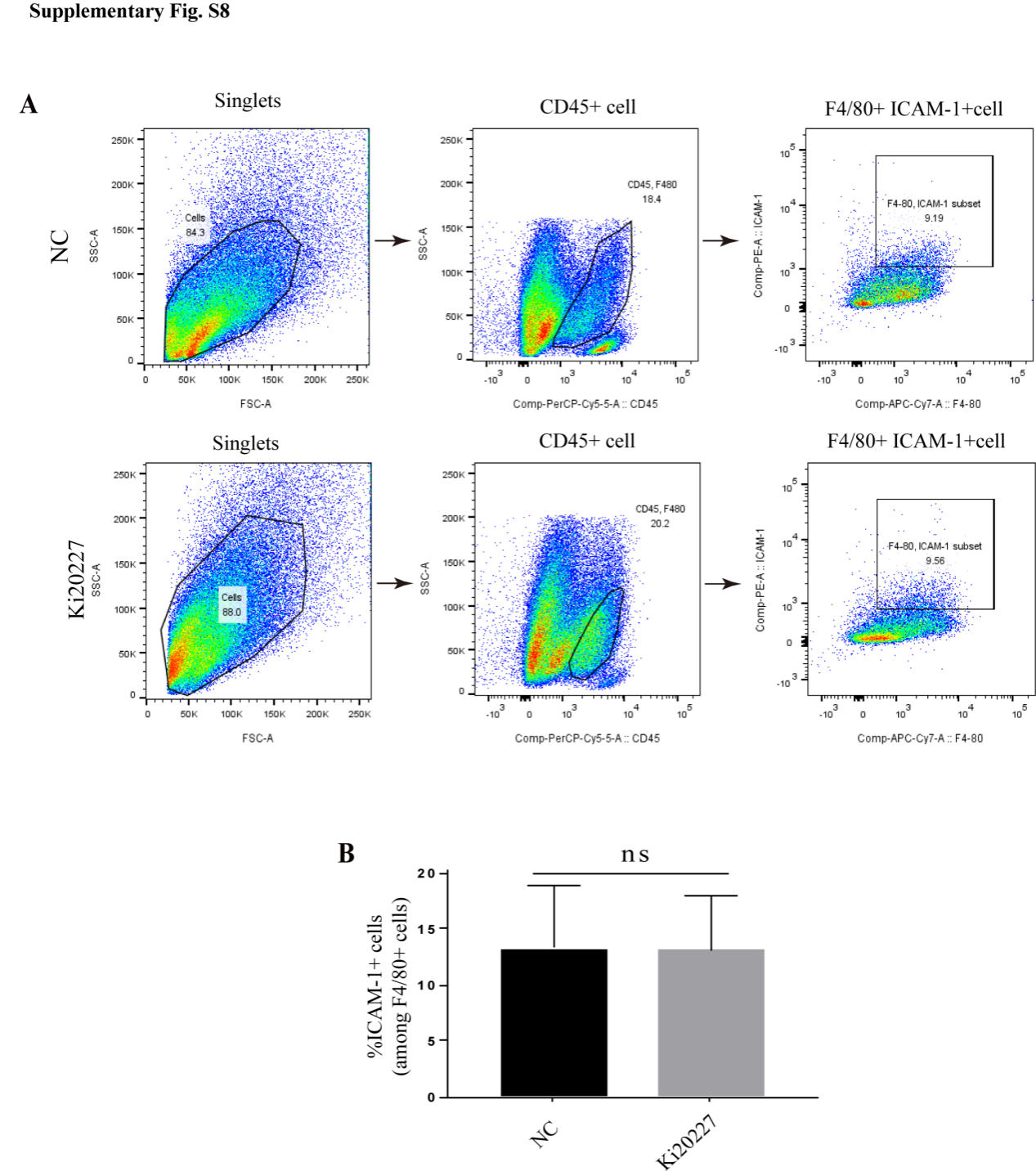


**Supplementary Figure 8.** Percentage of ICAM-1^+^ macrophages isolated from NC and Ki20227 BCa tissues was analyzed by flow cytometry and quantification.


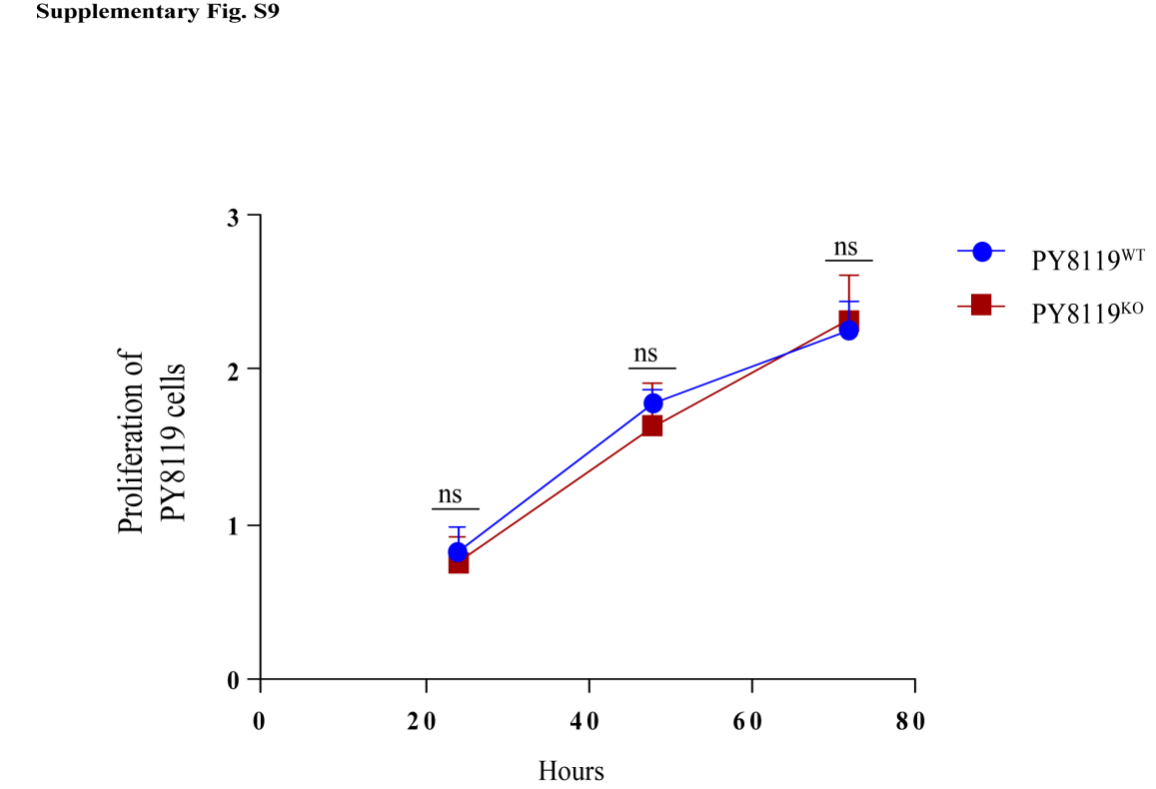


.**Supplementary Figure 9.** Quantification of cell viability between PY8119^KO^ cells and PY8119^WT^ cells.


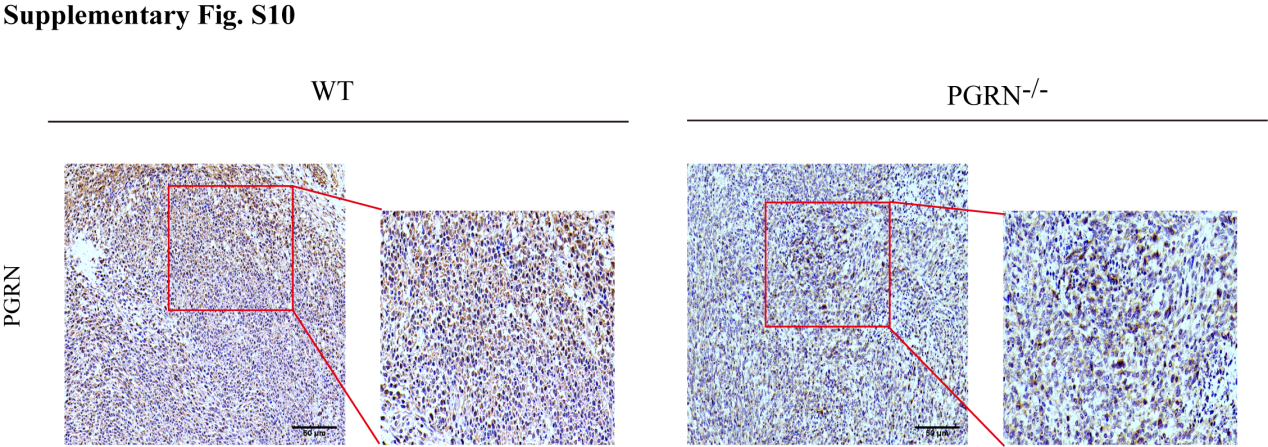


**Supplementary Figure 10. PGRN ^-/-^ group expressed low level of the PGRN in the mouse model.**

IHC images of PGRN^+^cells in tumors from WT and PGRN^-/-^mice; scale bar, 50um .


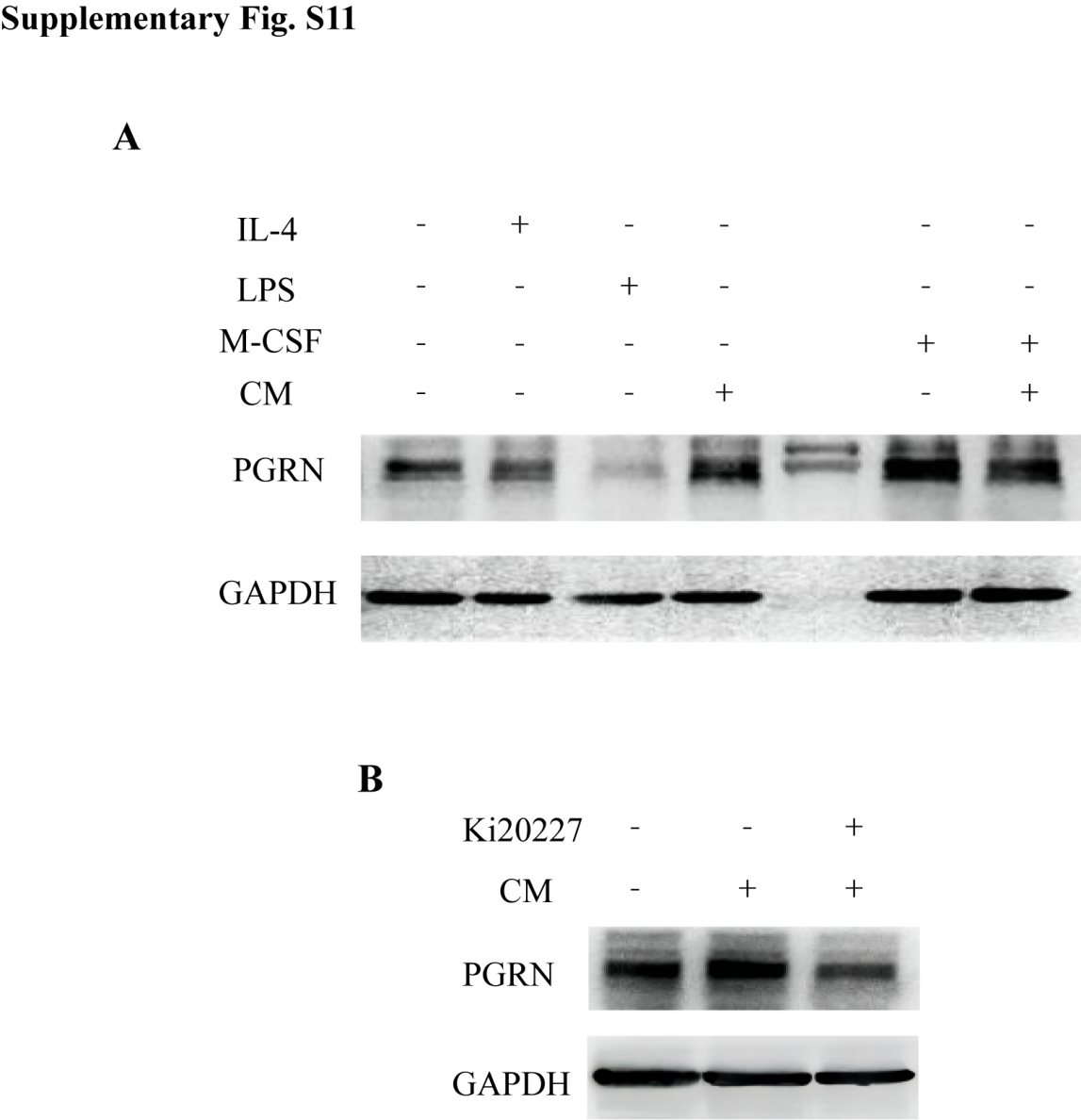


**Supplementary Figure 11. M-CSF can up-regulate PGRN expression of macrophages.g**

1. B**)** Western blot was performed to analyze PGRN expression in Raw267.4 cells.


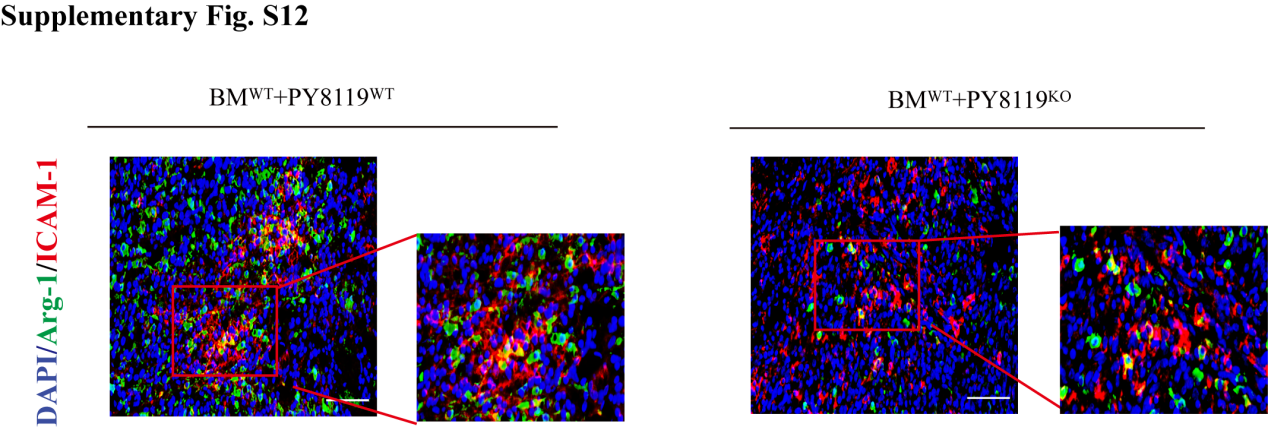


**Supplementary Figure 12. Macrophages expressed high level of the ICAM-1 in breast cancer.**

Representative images of MxIHC staining in tumor tissues; scale bar, 50um .


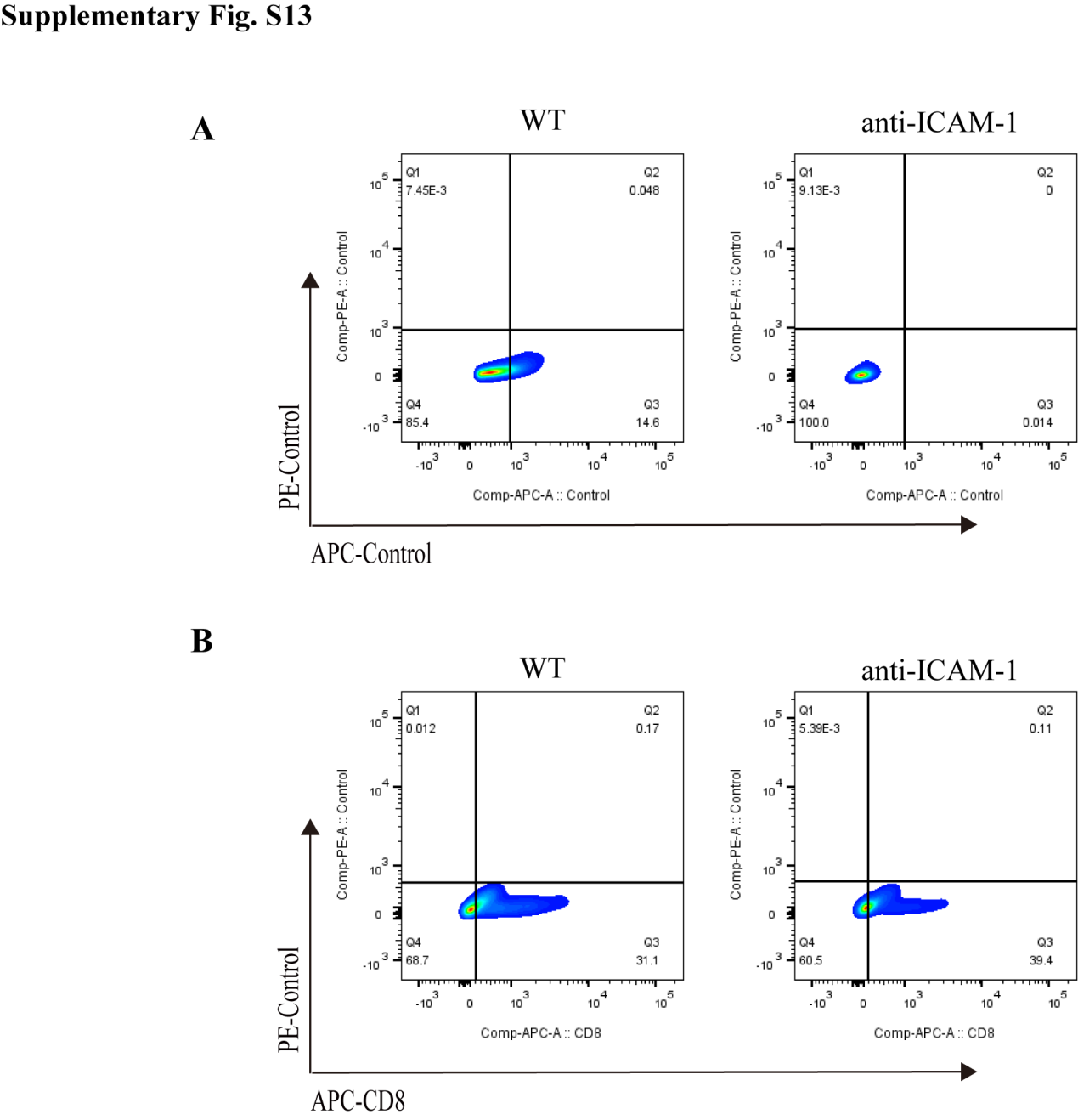


**Supplementary Figure 13. A,** The blank group for CD8^+^Ki67^+^T cell and CD8^+^ CD69^+^T cell (Fig 4D-E) in flow cytometry assays. **B,** The blank group for CD8^+^IFNγ^+^T cell (Fig 4F) in flow cytometry assays.
